# The Nature, Extent, and Consequences of Genetic Variation in the *opa* Repeats of *Notch* in *Drosophila*

**DOI:** 10.1534/g3.115.021659

**Published:** 2015-09-10

**Authors:** Clinton Rice, Danielle Beekman, Liping Liu, Albert Erives

**Affiliations:** Department of Biology, University of Iowa, Iowa City, Iowa 52242-1324

**Keywords:** polyglutamine, *Notch*, developmental genetics, DGRP, gene regulatory networks

## Abstract

Polyglutamine (pQ) tracts are abundant in proteins co-interacting on DNA. The lengths of these pQ tracts can modulate their interaction strengths. However, pQ tracts >40 residues are pathologically prone to amyloidogenic self-assembly. Here, we assess the extent and consequences of variation in the pQ-encoding *opa* repeats of *Notch* in *Drosophila melanogaster*. We use Sanger sequencing to genotype *opa* sequences ($5^{\prime }$-CAX repeats), which have resisted assembly using short sequence reads. While most sampled lines carry the major allele *opa31* encoding Q_13_HQ_17_ or the *opa32* allele encoding Q_13_HQ_18_, many lines carry rare alleles encoding pQ tracts >32 residues: *opa33a* (Q_14_HQ_18_), *opa33b* (Q_15_HQ_17_), *opa34* (Q_16_HQ_17_), *opa35a1*/*opa35a2* (Q_13_HQ_21_), *opa36* (Q_13_HQ_22_), and *opa37* (Q_13_HQ_23_). Only one rare allele encodes a tract <31 residues: *opa23* (Q_13_–Q_10_). This *opa23* allele shortens the pQ tract while simultaneously eliminating the interrupting histidine. We introgressed these *opa* variant alleles into common backgrounds and measured the frequency of *Notch*-type phenotypes. Homozygotes for the short and long *opa* alleles have defects in embryonic survival and sensory bristle organ patterning, and sometimes show wing notching. Consistent with functional differences between *Notch opa* variants, we find that a *scute* inversion carrying the rare *opa33b* allele suppresses the bristle patterning defect caused by *achaete*/*scute* insufficiency, while an equivalent *scute* inversion carrying *opa31* manifests the patterning defect. Our results demonstrate the existence of potent pQ variants of Notch and the need for long read genotyping of key repeat variables underlying gene regulatory networks.

Questions about the nature of polyglutamine (pQ) tracts in DNA-binding transcription factors and co-factors have arisen ever since the discovery of the *opa* ($5^{\prime }$-CAX) triplet repeats in the *Drosophila* gene encoding *Notch* (*N*) (Wharton *et al.* 1985). This was followed by similar reports of CAG-triplet repeats encoding pQ tracts in other regulatory genes of flies (Kassis *et al.* 1986), yeast (Pinkham *et al.* 1987; Suzuki *et al.* 1988; Bricmont *et al.* 1991; White *et al.* 1991) and other fungi (Yuan *et al.* 1991), mammals (Courey and Tjian 1988), and insect viruses (Carson *et al.* 1991). The significance and interest for human genetic disorders increased after pQ tract expansion was implicated in spinal cerebellar ataxias (SCAs), Huntington’s disease, and other neurodegenerative disorders (Biancalana *et al.* 1992; La Spada *et al.* 1992; Orr *et al.* 1993; Snell *et al.* 1993; Andrew *et al.* 1993).

Study of the Sp1 transcription factor led to the proposal that glutamine-rich regions are transactivation domains associated with co-interacting factors sensitive to *cis*-regulatory binding site spacing (Courey and Tjian 1988; Kadonaga *et al.* 1988; Courey *et al.* 1989) . Similarly, it was found that transcriptional enhancers integrating developmental morphogenic signals mediated by pQ-rich factors are sensitive to binding site spacing (Crocker *et al.* 2008, 2010; Crocker and Erives 2013). This led to a series of investigations that strongly implicated the selection of microsatellite repeat (MSR) variants in tuning enhancers targeted by pQ-rich transcription factors (Crocker *et al.* 2008, 2010; Brittain *et al.* 2014). Thus, we suspect that this MSR *cis*-enrichment at enhancers is a consequence of their being targeted by pQ-rich factors, which can also functionally evolve by MSR-related slippage in *trans*. Both *cis*-regulatory and *trans*-regulatory coding variation via MSR variants could affect the degree of pQ β-sheet interdigitation between the pQ tracts of TFs binding to adjacent sites in *cis*. Polyglutamine-rich factors have a tendency to aggregate (Scherzinger *et al.* 1997, 1999; Chen *et al.* 2002) but, more specifically, they may do so through the Perutz polar zipper (Perutz *et al.* 1993, 1994). The Perutz polar zipper is a strong β-sheet structure created by two adjacent proteins interdigitating together. Furthermore, polyglutamine-rich regions are frequently embedded in intrinsically disordered domains (Tóth-Petróczy *et al.* 2008), thereby reserving a role for a DNA enhancer scaffold to precipitate complex formation (Brittain *et al.* 2014).

To explore the natural relationship between pQ tract length and transcriptional interaction networks, we have focused on the Notch intracellular domain (NICD) for several reasons. First, this domain functions as a mostly dedicated coactivator of the highly conserved Su(H) transcription factor (Fortini and Artavanis-Tsakonas 1994; Furukawa *et al.* 1995; Lecourtois and Schweisguth 1995; Bailey and Posakony 1995). Second, Notch acts in a large number of developmental contexts involving tissue-specific enhancers targeted by Su(H) and other pQ-rich transcription factors. Notch pQ variants can thus be assayed through Notch-target reporter assays and other characterized assays (macrochaete patterning and SOP lineage specification) indicative of Notch activity. Third, NICD contains a single, long pQ tract interrupted only by a single histidine residue. The sequences from both the reference iso-1 (Adams 2000) and Canton-S (Kidd *et al.* 1986) strains feature the wild-type number of 31 consecutive 5′-CAX codon repeats in the eighth exon of *Notch*, the majority of which are 5′-CAG triplets. We refer to this *opa* repeat configuration of *Notch* as the *opa31* allelic type. The *opa31* version of *Notch* encodes the pQ tract, Q_13_HQ_17_. Additional work has shown the existence of a neutral $5^{\prime }$-CAG expansion in some *Notch* alleles corresponding to the *opa32* type encoding Q_13_HQ_18_ (Tautz 1989; Lyman and Young 1993).

Here, we sample the range of *opa* variants in the inbred isofemale Raleigh (RAL) lines constituting the *Drosophila melanogaster* genetic reference panel (DGRP), additional classical *Notch* alleles, X balancers, and other informative stocks. We find an extraordinary range of functionally variant *Notch opa* alleles that are invisible to current high-throughput genome sequencing and assembly methods. The distribution of *Notch opa* length variants is highly asymmetric for *D. melanogaster* and features a long tail of non-wild-type alleles in the range from 32 to 37 residues with unique alleles encoding the intervening histidine at distinct positions within the *opa* repeats. In stark contrast, we found no alleles in the range from 24 to 30 residues. Remarkably, a single deleterious allele, *opa23*, encodes an uninterrupted pQ tract of 23 residues. This suggests that the histidine may mitigate Notch pQ-seeded misfolding and/or aggregation, and that extreme shortening of the pQ tract can be balanced by simultaneous loss of the histidine. To determine the extent to which *Notch opa* variants are associated with *Notch* phenotypes, such as bristle patterning and wing notching defects, we introgressed RAL X chromosomes and X chromosomal regions differing in their *opa* repeat configuration into a common background. We find that both short and long extreme *opa* variants are significantly associated with aberrant embryonic levels of both full-length Notch and NICD, embryonic failure, bristle patterning defects, and occasionally wing notching. Our results also indicate that these phenotypes are frequently suppressed by genomic modifiers present in inbred stocks. We propose that the *Notch opa* repeat configuration is an important species-specific gene regulatory network variable.

## Materials and Methods

### PCR amplification

Genomic DNA was extracted from small pools of flies and individual flies of different lines. We used Invitrogen’s Platinum Taq High Fidelity, which is a mixture of: recombinant “Platinum” *Taq* DNA polymerase; a *Pyrococcus sp. GB-D* DNA polymerase with $3^{\prime }\to 5^{\prime }$ exonuclease proofreading activity; and a Platinum *Taq* antibody for hot starts.

#### Amplification of opa repeats:

Regions of *Notch* containing the *opa* repeats of exon 8 were amplified via conventional PCR thermocycling (30 cycles of 94° → *T_a_* → 72°) using one of the three following primer pairs and associated annealing temperatures (*T_a_*): OPA440f ($5^{\prime }$-CAG TCG CGA CCC AGT CTA C) and OPA440r ($5^{\prime }$-CCC GGA GAT CCA CAA AAT CCA) with a 58°*T_a_*, OPA808f ($5^{\prime }$-TTA CTT GTT ACA GGC TCG CCA TCG) and OPA808r ($5^{\prime }$-CCT CGC TCC AAT CGG AAT TCG) with a 67°*T_a_*, or OPA720-597f ($5^{\prime }$-CCG GCA ATG GAA ATA GCC ACG) and OPA720-597r ($5^{\prime }$-AGG GCG GAT TCA TTT GAC CCG) with a 54°*T_a_*.

#### Amplification of In(3R)K-only fragment:

To detect the In(3R) Kodani inversion, we used the following primers normally located on the same DNA strand: In(3R)K-R1/F1 ($5^{\prime }$-TCG AAG CCC GTG TGG TAA TC), which acts as forward primer if inversion present, and In(3R)K-R3 ($5^{\prime }$-TTC TCC CAA CGC ATC ACC AAA). We find that this primer pair amplifies a ∼1250 bp fragment if the inversion is present. When no inversion is present, amplification with R1 and In(3R)K-F2 ($5^{\prime }$-CTG GAC AGG AAG GGC GTC ATT AGC) gives a noticeably larger band than the inversion band (see Supporting Information, File S1).

### Cloning and sequencing

PCR products were purified by gel electrophoresis (1% agarose gel), excision of the band, and gel purification (QIAquick Gel Extraction Kit, Qiagen). Purified PCR products were ligated into the pGEM-T Easy plasmid (Promega) and transformed into JM109 competent *Escherichia coli* (Promega) according to standard protocols. Blue/white screening was used to identify colonies with plasmids carrying inserts. Cells from individual white colonies were grown in Luria Broth with ampicillin overnight. Plasmids were isolated from cultures using the QIAPrep Spin Miniprep Kit (Qiagen) and sequenced (ABI BigDye3.1 and ABI 3730, Applied Biosystems) with T7, SP6, M13, and/or *Notch*-specific PCR primers. The RAL lines and *sc* inversion lines were genotyped by sequencing 7–10 independent clones with most lines having at least 10 clones and some lines having hundreds of clones sequenced as part of quality control (QC) experiments. The classical *Notch* alleles, X balancers, the wopa23, RAL-100, and RAL-105 lines were genotyped by sequencing three to seven clones, and many were also confirmed by sequencing a PCR-amplified genomic DNA.

### QC experiments for opa genotyping

To ensure true genotypes could be distinguished from variant clones produced by somatic mutation, PCR mutagenesis, and/or interculture and intraculture environmental contamination, we conducted the following quality control experiments.

To gauge the extent of PCR error, we conducted eight replicate PCR reactions using as genomic template the miniprep DNA from one *opa31* clone. We obtained sequences for two to three clones from each reaction for a total of 21 sequenced clones. Except for one sequenced clone from a reaction with three sequenced clones, all were of the original genotype. The single exception corresponded to an *opa30L* contraction (Q${}_{12}$HQ${}_{17}$) on the left (L) side of the histidine codon, a PCR contraction error rate of approximately 4.8%. Similar contraction error rates are seen in our other QC experiments.

To gauge the extent of somatic variation, we used PCR primer pair OPA720-597f/r to clone and sequence 218 long (720 bp) and 276 short (597 bp) *Notch* clones from BDGP stock #26820, which contains a *P*-element carrying a *UAS-N-full* cDNA that is presumably nonfunctional in the absence of a GAL4 driver. Both endogenous and transgenic sequences feature a (Canton-S) wild-type opa31*3 genotype (shortened to *opa31* here). Of the 218 endogenous clones, 94.0% were of the original *opa31* genotype (205 clones), and 6.0% were contraction variants (nine *opa30L* clones, one *opa29L* clone, and two *opa30R* clones). Of the 276 nonfunctional cDNA clones that we sequenced, 94.2% were of the original *opa31* genotype (260 clones) and 5.8% were contraction variants (13 *opa30L* clones, two *opa29L* clones, and one *opa27R* clone). Thus, both the functional endogenous locus and the presumably nonfunctional transgenic loci have identical error rates in a similar range as the PCR-based experiment.

To gauge the extent of intraculture environmental contamination, we sequenced 360 clones from 39 individual larvae and pupae from the RAL-142 stock, which we initially identified to be polymorphic for *opa31* and *opa32* in equal amounts. We sequenced 5–13 clones per RAL-142 individual with an average of 9.2 clones per individual. Of the 360 clones, 93.6% were of the original genotypes (337 clones of either *opa31* or *opa32*), 5.8% were contraction variants (10 clones each of either *opa30L* or *opa31L*, and one clone of *opa28L*), and 0.6% corresponded to one *opa33L* expansion variant encoding Q${}_{14}$HQ${}_{18}$ (two clones). We measured a 144:124 female-to-male sex ratio in this stock (53.7% female) and thus estimated observing 14 flies for each of *opa31*-only and *opa32*-only genotypes (males and females) and 11 heterozygous genotypes (*opa31*/*opa32* females) under Hardy-Weinberg equilibrium. We observed close to the predicted numbers with 13 *opa31*-only genotypes, 14 *opa32*-only genotypes, and 12 *opa31*/*opa32* heterozygotes if we assume that all individuals with clonal genotype frequencies of one singleton “minor” allele in eight or more sequenced clones are the result of environmental contamination from the DNA of bottle mates of other genotypes. Because there was a statistically impossible number of such true genotype calls, we concluded that these numbers indicated an intraculture environmental contamination rate of 5.7%.

To gauge whether the shortened *opa23* allele in the RAL-646 line has an attenuated or otherwise aberrant mutational rate, we sequenced 358 clones from 38 individual larvae and pupae from this line. These individuals were picked on the same day as the RAL-142 individuals described above using the same picking tool wiped with ethanol between picks. Of the 358 sequenced RAL-646 clones, we identified six and seven clones corresponding to *opa31* and *opa32* alleles, respectively. These correspond to the two RAL-142 alleles, and an interculture contamination rate of 3.6%. These 13 clones were minor contaminants in 10 of the 38 RAL-646 individuals, all of which were found to be homozygous for *opa23*. Although this low rate of contamination did not prevent genotyping of any of the 38 individuals, we subsequently adopted more stringent control of reagents and picking tools and eliminated environmental contamination in later *opa* sequencing experiments. QC experiments three and four were conducted at the start of this project and corresponded to the only time we ever saw evidence of environmental contamination. Putting aside the 13 *opa31*/*opa32* contaminant clones, we were left with 340 total clones sequenced from this stock. Of these, 96.5% were of the original *opa23* genotype (333 clones) and 3.5% were *opa22L* contractions (12). In summary, the contraction variants were identical to the (CAG)_7_ → (CAG)_6_ contractions seen in all the other QC experiments, including the PCR control.

### Reassembly of RAL-646 opa repeats

To understand the discrepancy in the DGRP “Line-646” assembly for RAL-646, which was reported as a single H → Q variant but which we found to be a 24-bp deletion, we asked whether this line had changed since the time of the DGRP sequencing and assembly. For example, it could have been the case that an original change of the intervening His to Gln resulted in a toxic uninterrupted pQ tract. This nonsynonymous mutated allele could have been replaced in the stock by a subsequent more advantageous deletion that shortened this tract. To address this question, we obtained all of the original DGRP sequencing reads and aligned them to the iso-1 reference alignment (File S1). Many of the reads in this alignment had mismatches to each other and to the reference sequence, and a few only had one or two that were plausibly polymorphisms specific to this stock. After removing the reads with high levels of mismatch, we are left with an alignment that suggests the two synonymous changes and the indicated nonsynonymous change seen in the Line-646 DGRP assemblies. Nonetheless, when we reassemble the original DGRP reads by alignment to our long Sanger sequence, we increase the number of original DGRP reads with 100% alignment (File S2). Thus, the original RAL-646 stock was and continues to be homozygous for the unique *opa23* allele and not the reported substitution variant encoding an H → Q *opa31*.

### DGRP line outcrossing

To produce our outcrossed lines, one to five virgin RAL females (from RAL-# parent stock lines) were crossed with two to three X balancer males from lab stocks of *FM7c*/*N${}^{1}$* (background one) or *FM7a* (background two). We performed five generations of outcrossing of virgin females to the balancer stock males, followed by two generations of inbreeding to rehomozygose the RAL X chromosome. We refer to these lines as the RALX#-bg1 and RALX#-bg2 outcrossed lines.

To produce the *w*^1118^ *N^opa23^* line (“wopa23”), we first crossed RAL-646 (*opa23*) virgin females with *w*^1118^ males (P${}_{0}$). Second, we took heterozygous F${}_{1}$ virgin females and crossed them to *FM7c* males. Based on the distance between *w* and *N* and published recombination rates for this region, we expected a recombination rate of 1% (Comeron *et al.* 2012). So, third, we took 202 individual white-eyed F_2_ males, choosing as much as possible those that had ectopic macrochaetes, and set up individual crosses with *FM7a* virgin females. Fourth, after F_3_ larvae were visible, we then genotyped the *opa* repeats from the single F_2_ males by Sanger sequencing of amplified genomic DNA. Fifth, after approximately 50 genotypes, we genotyped our first white-eyed *opa23* male from one of the F_2_ crosses. We took the F_3_ virgin females and crossed them to *FM7a* sibling males. Finally, we crossed the non-Bar, white-eyed F_4_ males to heterozygous sibling females and selected only white-eyed nonbalancer flies in the F_5_ generation to homozygose the recombined *w opa23* X chromosome.

### Embryonic survival assays

Populations of 400–600 parent flies from individual DGRP parent stocks (RAL-# lines) or outcrossed lines (RALX#-bg1 and RALX#-bg2) were raised at room temperature (22–24°) and allowed to lay eggs on a blue-dyed apple juice (AJ) agar plate at 25° for 4 hr 1–12 d after eclosion. Efforts were made to standardize the size of the parent population, as well as the age spectra. Embryos were counted in sections of the AJ agar plates so that similar numbers and densities of embryos were assessed across lines. These sections were placed in the middle of larger AJ agar plates with yeast paste smeared around the outer portion of the plate to attract larvae away from the middle. The fraction of embryos failing to hatch was determined by counting the number of unhatched embryos approximately 40 hr after the end of the laying period.

### Macrochaete scoring

The ectopic macrochaete phenotypic data shown in Figure 5A involved scoring doubled (nonsplit) anterior and posterior dorsocentral macrochaetes (DCs), anterior and posterior scutellar macrochaetes (SCs), and posterior postalars (pPAs).

The macrochaete frequency plots for the 13 macrochaetes of the hemi-notum involved the following scoring system applied to each of the 13 macrochaete locations. A score of 1.0 was assigned for a normal-looking macrochaete (normal thick bristle) at a correct location. A score of 0.0 was assigned for a missing bristle, and the presence of an empty socket was recorded if observed. A score of 2.0 was assigned if an ectopic bristle was observed near a normal position, or if a split bristle was observed (two bristles emanating from a single socket). Split bristles were rare and such cases were recorded if observed. A score of 0.5 or other fractional values were assigned for abnormally slender bristles that were either larger than a microchaete or emanating from a robust macrochaete-type socket. Occasionally, an ectopic bristle was observed exactly mid-distance from the anterior and posterior dorsocentrals or scutellars, and in such cases each bristle position was assigned a value of 1.5. A score of −1.0 (1.0–2 defects = −1.0) was assigned when an empty or missing socket at a correct location was observed together with a nearby ectopic macrochaete. Rarely, a score of −2.0 (1.0–3 defects = −2.0) was assigned when two empty sockets replaced a single normal macrochaete. The data for a specific bristle position for a given genotype seldom involved *both* ectopic (>1.0 scores) and missing bristles (<1.0 scores). To prevent the cancellation or masking of such defects using the described scoring system, only the more frequent defect (ectopic or missing bristle, but not both) was scored, with the data for the less frequent defect being assigned 1s. Typically, 40–60 hemi-nota from 20–30 adult flies of each sex were scored, but more were scored when many of the flies had wild-type macrochaete patterning.

### Western immunoblots

Lysates were obtained from 50 μL of embryos collected on AJ plates and aged to either 0–2 hr or 4–6 hr after egg deposition (AED). SDS-PAGE (BIO-RAD TGX acrylamide) gels were loaded with 8 μL of protein extracts in loading buffer and typically run for 0.5 hr at 206 V using the BIO-RAD casting system and electrophoresis apparatus (Mini-PROTEAN Tetra Cell). Gel-separated proteins were transferred to PVDF blotting paper and blocked overnight at 4° with 5% nonfat milk in PBT (1× PBS + 0.1% tween-80). Blots were then incubated with one or two of the primary antibodies diluted 1:500 in PBT-1.0 (1× PBS + 1% Tween-80) for 1 hr of shaking at room temperature (RT). Mouse C17.9C6 (Fehon *et al.* 1990) and E7 monoclonal antibodies, which are specific for *Drosophila* Notch intracellular domain and β-tubulin, respectively, were obtained from the Developmental Studies Hybridoma Bank (University of Iowa) and used as primary antibodies. After four washes in 1× PBT (15 min per wash), the blots were incubated 1 hr at RT shaking with HRP-conjugated goat anti-mouse secondary antibodies at a 1:10,000 dilution. PVDF paper was washed four times in PBT (15 min per wash) before being incubated with ECL detection solutions per manufacturer’s instructions. The blot paper was then inserted in a plastic envelope and exposed to film.

### Data availability

All of the RALX-bg1 and RALX-bg2 strains, which feature RAL X chromosomes introgressed into two common backgrounds, are available on request, as are the wopa23 lines carrying *w*^1118^, *N^opa23^*. Representative RALX-bg1 samples of three *opa* variants are also available from the Bloomington stock center under the following stock numbers: #63522 for *opa23* (RALX646-bg1); #63523 for *opa31* (RALX440-bg1); and #63524 for *opa35* (RALX237-bg1). These were chosen for showing the least overall embryonic failure (see File S3).

File S1 is a figure showing genotyping results for the Kodani inversion. File S2 contains the reassemblies of the original sequencing reads for the *opa* repeats of *Notch* from the DGRP Line-646. File S3 is a figure showing the separate embryonic assay results for the RALX-bg1 and RALX-bg2 series.

## Results

### Nomenclature for the many opa variations of Notch

**Figure 1:**
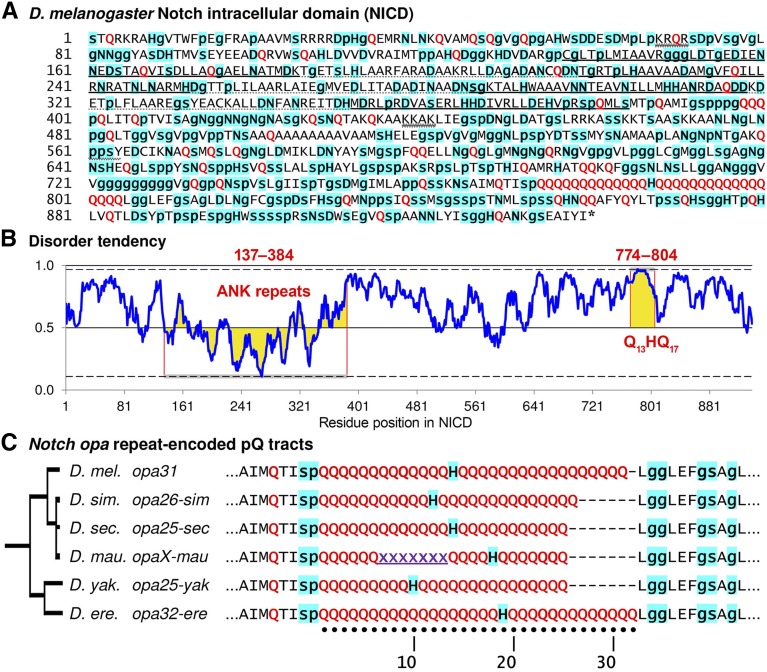
The cleaved Notch intracellular domain (NICD) is characterized by a polyglutamine (pQ) tract configuration that is unique to each *Drosophila* species. (A) Shown is the 939-residue-long polypeptide sequence for NICD from *D. melanogaster*. Residues that tend to be secondary structure breakers are highlighted in cyan (p, g, s, D, N, and H), with the smaller amino acids shown in lowercase lettering. Glutamines (Qs) are shown in red. The seven ankyrin repeats are shown in alternating bold and dotted underlining. Two nuclear localization sequences, NLS1 (KRQR) and NLS2 (KKAK), are indicated with double wavy underlining. The Nedd4 ubiquitination site (ppsY) is also indicated in light wavy underlining. (B) Much of the NICD is disordered, as demonstrated by this plot of long disorder tendency based on pairwise energy content (IUPred) (Dosztányi *et al.* 2005a,b). The ankyrin (ANK) repeats (residue positions 137–384) and the pQ tracts (Q_13_HQ_17_ at residue positions 774–804) are notable for having the least and most disorder tendencies, respectively (highlighted in yellow). (C) The *opa*-encoded pQ repeats of the *melanogaster* subgroup are characterized by two adjacent pQ tracts separated by a single conserved histidine. While the surrounding amino acids are conserved, each species features a unique pQ configuration characterized by the length of the pQ tracts on either side of the histidine. The assembly for *Drosophila mauritiana* indicates a unique position for the His residue even though the entire *opa* repeat sequence is still uncertain (Garrigan *et al.* 2012). In this study, we refer to different *Notch opa* configurations by appending the length suffix to the word “opa.” We add an additional index when more than one configuration exists for a given length as distinguished by the position of the single histidine (*e.g.*, *opa25-sec* *vs.* *opa25-yak*, or *opa33a* *vs.* *opa33b* in *D. melanogaster*). Unless a species is indicated (*e.g.*, *opa26-sim*), all *opa* designations refer to alleles from *D. melanogaster*.

The *opa* nucleotide repeats are located in the eighth exon of *Notch* (*N*) and encode two polyglutamine (pQ) tracts separated by a single histidine (H) (Figure 1). This pQ-H-pQ sequence is part of the Notch intracellular domain (NICD), which is translocated into the nucleus upon cleavage (Figure 1A). These pQ tracts are intrinsically disordered and surrounded by many other similar peaks of disorder (Figure 1B). While the NICD pQ tracts are immediately flanked by conserved amino acid sequence, we find evidence of multiple *opa* repeat configurations that are unique to the *Notch* gene of *D. melanogaster* (Figure 1C). To better relate our phenotypic results from studying variant *Notch opa* alleles, we introduce a simple nomenclature for the different *opa*-encoded pQ configurations.

The single intervening histidine (His, H) is relevant to our findings and is a convenient marker for naming distinct *opa* configurations even when they share the same number of repeats. The His codon ($5^{\prime }$-CAY) is closely related to the codon for glutamine (Gln, Q) ($5^{\prime }$-CAR), and so one might expect to frequently observe His codon turnover within this tract, but this is not the case. The His codon changes position only indirectly after changes in the lengths of the two flanking pQ tracts. Thus, it is likely that the single His residue is highly conserved for a specific role. For example, His residues are known β-sheet breakers and have been found to attenuate the β-sheet forming potential of pQ peptides (Sharma *et al.* 1999; Sen *et al.* 2003; Kim 2013). Interestingly, species that are closely related to *D. melanogaster* each possess a single His residue at a unique position within the pQ tract (Figure 1C).

**Figure 2:**
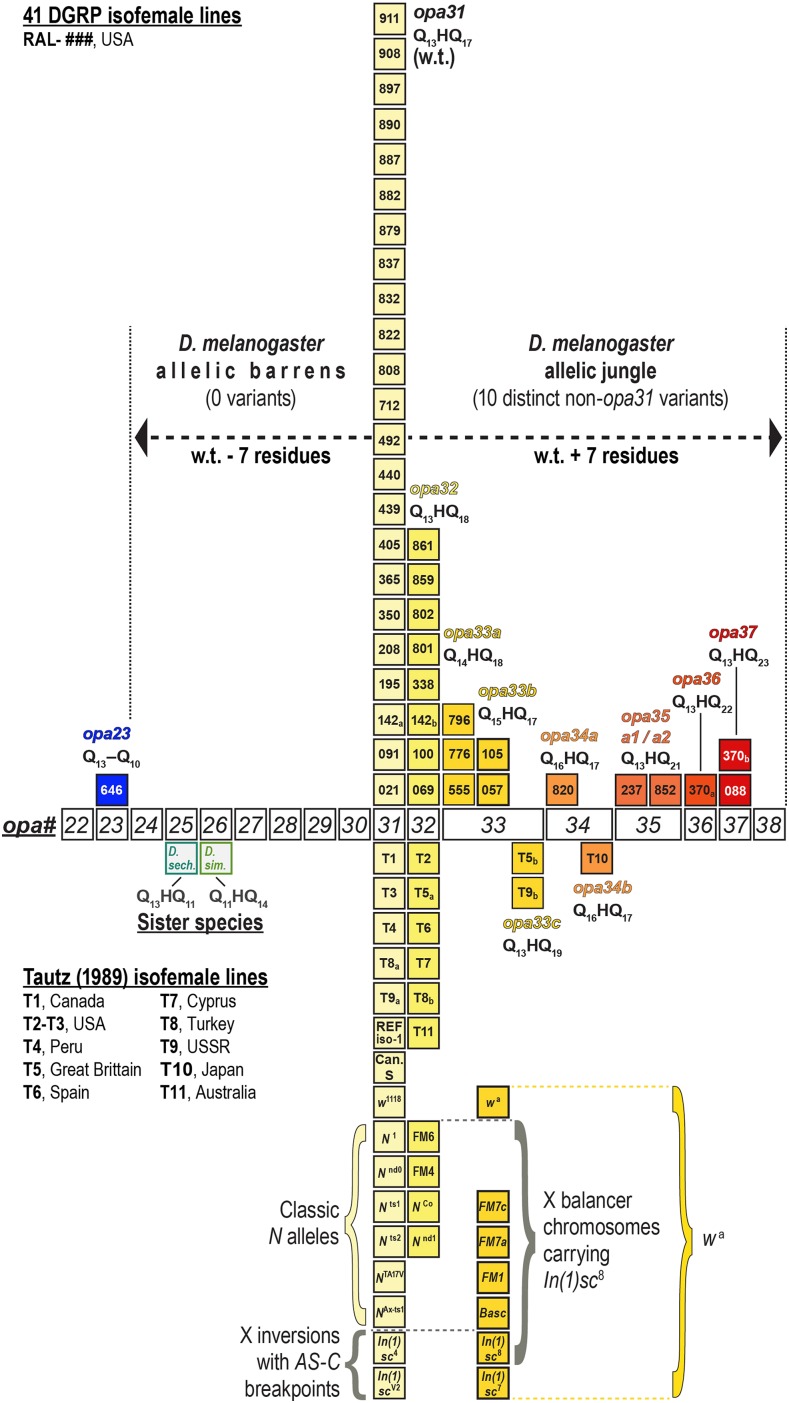
Shown is the distribution of *opa*-encoded pQ tracts of 41 RAL lines (boxes above line) genotyped by Sanger sequencing of multiple independent clones. Variants are shown from short (left, bluer boxes) to long (right, redder boxes), with each column representing a distinct *opa* allele. Heterozygous haplotypes (142a/142b and 370a/370b) are each represented by separate boxes. The distribution of *opa* alleles from *D. melanogaster* is highly asymmetric and does not harbor any alleles encoding <31 residues except for *opa23*, which is much shorter and missing the His codon. Because of this asymmetry, we refer to the range *opa24*–*opa30* as the *D. melanogaster* allelic barrens, and to the range *opa32*–*opa38* as the allelic jungle. Shown below the *opa-#* axis are several classic *Notch* alleles, X balancers, and X inversions, which we also genotyped (see text). Canton-S wild-type and *N^Co^* and *N^nd^*^1^ mutant alleles have been previously reported (Kidd *et al.* 1986; Lyman and Young 1993). Also shown is the distribution of *opa* genotypes for 11 isofemale lines from different worldwide populations (Tautz 1989).

**Figure 3:**
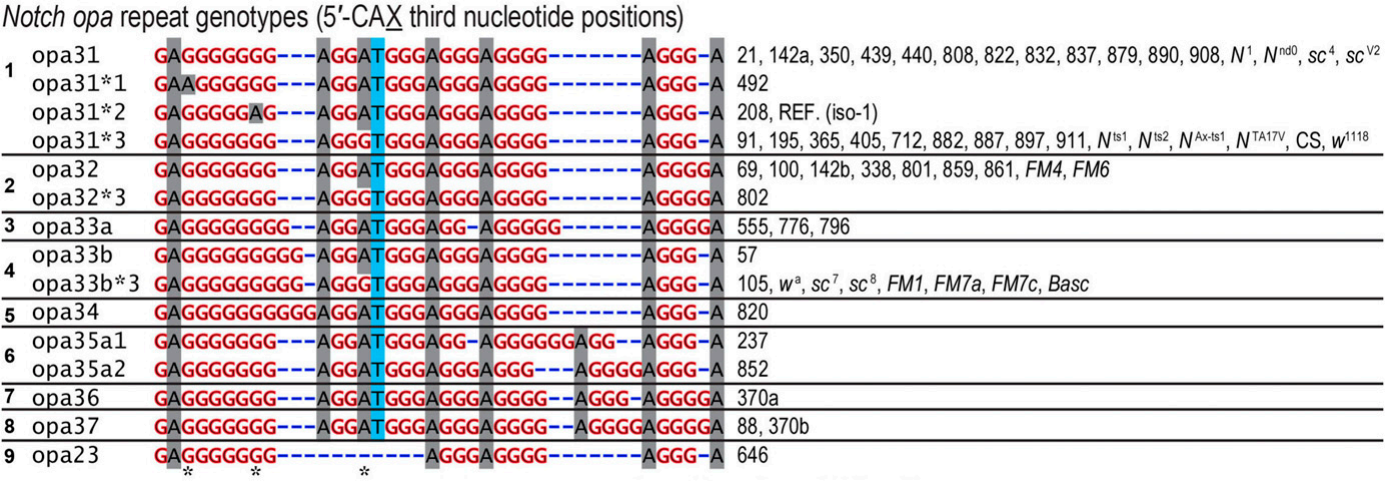
Diverse RAL nucleotide sequences for the *opa* repeats of *D. melanogaster* encode nine distinct pQ-isoforms of Notch. The *opa* repeat genotypes (third codon positions of $5^{\prime }$-CAX shown) also demonstrate that the longest repeats of $5^{\prime }$-CAG coincide with the most polymorphic regions, consistent with the effect of repeat length on replication slippage frequency and nucleotide substitution rates. We also note that the $3^{\prime }$ region of the *opa* repeats corresponds to a repeat of repeats, (GGGGA)_2-3_, and derivatives thereof. This region is also a hypervariable site. Asterisks indicate three positions where synonymous substitutions were observed. Canton-S (CS) and other genotyped stocks are also listed. BDGP stock #26820, which is not listed, carries a UAS-N-FULL cDNA featuring a stable opa31*3 nucleotide sequence as determined in QC experiments (see *Materials and Methods*).

To distinguish the variants by repeat length, we refer to each *Notch opa* configuration by a length designation. For example, here *opa31* will refer to the *opa* nucleotide repeats encoding the 31 residues Q_13_HQ_17_. For cases where there were two or more observed allelic classes of the same length, we use an additional lowercase suffix letter. For example, here *opa33a* will designate Q_14_HQ_18_ and *opa33b* will designate Q_15_HQ_17_ (see Figure 2). For cases where identical pQ configurations are encoded by independently derived nucleotide sequences as determined by the unique patterns of $5^{\prime }$-CAG and $5^{\prime }$-CAA codons, we append an additional number to the letter suffix. Thus, for example, *opa35a1* and *opa35a2* both encode Q_13_HQ_21_ but have been derived by independent histories of insertions and deletions (see Figure 3).

The most frequent *opa* alleles, wild-type *opa31* and *opa32*, also have the most derived variants caused by single synonymous substitutions at the nucleotide sequence level. We will usually not distinguish between these and refer to them nonspecifically with an asterisk (opa31*) only when discussing features at the nucleotide level. (In this study we noted three such polymorphisms and refer to them by applying the suffixes *1, *2, and *3 to the *opa* genotype base name).

### Notch opa variants from the Raleigh lines (DGRP)

To sample a natural distribution of *Notch opa* alleles, we made use of the 205 inbred isofemale lines constituting the *Drosophila melanogaster* Genetic Reference Panel (DGRP) (Mackay *et al.* 2012). The DGRP stocks were founded by 1500 mated females of *D. melanogaster* caught in 2003 outside of a farmers market in Raleigh (RAL), North Carolina, and individually subjected to 20 generations of full-sibling inbreeding (Mackay *et al.* 2012). The initial bulk set of DGRP genome assemblies were based on paired short reads (Illumina 75 nucleotides), which are incapable of individually spanning the 93 base pairs constituting the typical *Notch opa* sequence and resolving alignment of its repeat structures. A subset of these was subsequently complemented with 454 reads as well as various consensus assembly strategies (Mackay *et al.* 2012; Huang *et al.* 2014). The DGRP Freeze 1.0 assemblies focused on identifying high-quality SNPs with significant minor allele frequencies (Huang *et al.* 2014). Subsequent reassemblies were used to establish an assembly consensus to improve the sequence genotyping around microsatellite repeat structures (Huang *et al.* 2014). However, in our experience with Sanger resequencing of the *Notch opa* repeats, the DGRP 1.0, and similar DGRP 2.0 assemblies are unreliable at this locus (Table 1). For example, of the 158 prefreeze PopDrowser assemblies based only on Illumina reads (Ràmia *et al.* 2012), more than 40% of *opa* sequences contain ambiguous base calls, particularly $5^{\prime }$-CAR codons. Similarly, of the 162 DGRP Freeze 1.0 assemblies based only on Illumina reads and aligned using different methods (Mackay *et al.* 2012), 33% of *opa* sequences contain ambiguous base calls.

**Table 1:** Sampling the *Notch opa* repeat genotype via Sanger sequencing

Group	% Non-*opa31**^a^*	Avg. Deviation*^b^*	RAL Lines (*Notch opa* genotype)
Notch Dirty Dozen*^c^*	58.3% (7/12)	1.7 a.a.	21 (*opa31*), 237 (*opa35a1*), 350 (*opa31*), 439 (*opa31*), 440 (*opa31*), 555 (*opa33a*), 646 (*opa23*), 776 (*opa33a*), 796 (*opa33a*), 801 (*opa32*), 802 (*opa32*), 822 (*opa31*)
Radiation-resistant*^d^*	58.3% (10.5/18)	1.8 a.a.	91 (*opa31*), 69 (*opa32*), 338 (*opa32*), 208 (*opa31*), 57 (*opa33b*), 492 (*opa31*), 142 (*opa31*/*32*), 879 (*opa31*), 370 (*opa36**^e^*/*opa37*), 808 (*opa31*), 237 (*opa35a1*), 88 (*opa37*), 405 (*opa31*), 646 (*opa23*), 801 (*opa32*), 105 (*opa33b*), 776 (*opa33a*), 195*^f^* (*opa31*)
Radiation Sensitive*^g^*	31.8% (7/22)	0.6 a.a.	21 (*opa31*), 350 (*opa31*), 365 (*opa31*), 439 (*opa31*), 440 (*opa31*), 555 (*opa33a*), 712 (*opa31*), 796 (*opa33a*), 802 (*opa32*), 820 (*opa34*), 822 (*opa31*), 832 (*opa31*), 837 (*opa31*), 852 (*opa35a2*), 859 (*opa32*), 861 (*opa32*), 882 (*opa31*), 887 (*opa31*), 890 (*opa31*), 897 (*opa31*), 908 (*opa31*), 911 (*opa31*)
*In(3R)K*/*In(3R)K**^h^*	100.0% (3/3)	3.7 a.a.	100 (*opa32*), 105 (*opa33b*), 646 (*opa23*)

a Each haplotype in the heterozygous RAL-142 and RAL-370 stocks counted as 0.5.

b Average absolute value of deviation from a wild-type length of 31 amino acids (a.a.).

c DGRP1.0 lines with ambiguous assembly sequence (contains Ns) or His → Gln (RAL-646).

d Top radiation-resistant RAL lines genotyped in order of average survival (Vaisnav *et al.* 2014): 91 (49.0/50 flies); 69 (47.5/50 flies); 338 (45.0/50 flies); 208 (44.5/50 flies); 57 (43.0/50 flies); 492 (42.0/50 flies); 142 (41.0/50 flies); 879 (39.0/50 flies); 370 (49/50 flies); 808 (31.5/50 flies); 237 (28.5/50 flies); 88 (26.5/50 flies); 405 (26.0/50 flies); 646 (9.5/50 flies); 801 (4.5/50 flies); 105 (2.0/50 flies); 776 (2.0/50 flies); 195 (2.0/50 flies).

e The *opa36* variant was observed only once in three sequences from a pool of three males and not observed in sequences from three individual males (total of 1/8 sequences).

f By virtue of a nonsynonymous substitution, this RAL line uniquely has a Q_7_ Twist, whereas other lines have a Q_2_LQ_4_.

g Radiation-sensitive RAL lines genotyped [0/50 flies surviving (Vaisnav *et al.* 2014)].

h Only three RAL lines are homozygous for Kodani inversion (Huang *et al.* 2014).

The difficulty of aligning lengthy repeat structures is compounded by the alternate subalignments made possible by the repeat structure (Huang *et al.* 2014). This difficulty is exacerbated at the *Notch opa* region when reads are aligned to the reference iso-1 assembly (isogenic strain with genotype *y*; *cn bw sp*) because it contains a minor synonymous polymorphism ($5^{\prime }$-CAG$\to 5^{\prime }$-CAA; see Figure 3) (Brizuela *et al.* 1994; Adams 2000).

We performed Sanger sequencing of several groups of DGRP lines, which we chose by various strategies (Table 1). Given the DGRP1.0 and DGRP2.0 sequences, we had a prior expectation of finding few length variants different from *opa31*. We therefore chose to sequence 11 lines, which had ambiguous sequence annotated in their *Notch opa* assemblies, and one line (RAL-646), which was annotated as a simple coding substitution changing the central histidine to glutamine. We find that this set of RAL stocks, which we refer to as the Notch Dirty Dozen, carried three previously unreported alleles: *opa23* (encoding Q_23_), *opa33a* (encoding Q_14_HQ_18_), and *opa35a1* (encoding Q_13_HQ_21_) (Figure 2, Table 1).

To assess the distribution of *opa* variants in DGRP lines with unambiguous assemblies, we obtained and genotyped 24 additional lines chosen as follows. A recent report on radiation-resistant RAL lines (Vaisnav *et al.* 2014) listed four of the Notch Dirty Dozen lines, which we had found to carry non-*opa31* variants, as exhibiting different degrees of radiation resistance (Table 1). We speculate that extremely variant *opa* lengths may lead to proteostatic stress. Aberrant pQ-related aggregation might induce the activity of stress response pathways and prime such lines to resist lethal doses of radiation (more on the phenotypes of *opa* variants later). To test this hypothesis, we genotyped 12 of the top radiation resistant lines (66/161) and 12 randomly chosen radiation-sensitive lines (within the range RAL-820–RAL-911), which constitute the bulk (∼60%) of the DGRP stocks (95/161). Altogether, we found that 58.3% of the radiation-resistant lines have non-*opa31 Notch* genotypes *vs.* 31.8% of the radiation-sensitive lines (Table 1). Furthermore, the average absolute value of deviation from the wild-type length of 31 amino acids is 1.8 amino acids for the radiation-resistant lines, whereas it is 0.6 amino acids for the radiation-sensitive lines (Table 1). Thus, nonwild-type *Notch opa* length variants are correlated to radiation-resistance.

The strange RAL-646 stock, which is homozygous for *opa23 Notch* on the X chromosome, is one of only three RAL lines that are also homozygous for the Kodani inversion *In(3R)K*, located in the right arm of chromosome 3 (Huang *et al.* 2014). Ten additional lines are heterozygous for *In(3R)K*, including RAL-440, which we genotyped as having wild-type (*opa31*) repeats. The Kodani inversion is a rare but cosmopolitan inversion that originated approximately 60,000–90,000 years ago, although it was recently discovered that it is almost entirely fixed in the African samples from the Oku range in Cameroon (Corbett-Detig and Hartl 2012). Because of the interesting *opa23*-related phenotypes, we genotyped the *opa* repeats of the two other lines that are also homozygous for the Kodani 3R inversion as originally described (Huang *et al.* 2014), RAL-100, and RAL-105 (Kodani inversions confirmed for all three in Figure S1). We found that RAL-100 is homozygous for *opa32*, whereas RAL-105 is homozygous for *opa33b*.

In Figure 2, we show all of the 41 RAL lines we genotyped as a single distribution. This distribution shows the frequencies of 10 different Notch *opa* isoforms sampled in the Notch Dirty Dozen (ambiguous DGRP1.0 sequence information), 12 additional radiation-resistant lines, 12 additional radiation-sensitive lines, and two additional lines homozygous for *In(3R)K* (individual lines per group are listed in Table 1). This distribution has three remarkable features. First, approximately 48% of examined RAL lines (19.5/41) do not have wild-type *opa31* repeat haplotypes and are thus not being accurately genotyped by current high-throughput methods. Second, it shows that there is a long diminishing tail of many rare *opa* expansion alleles in the *opa32–opa37* range, which we refer to as the “allelic jungle.” Third, in contrast to the long tail of rare expanded alleles, there is a relative deficit of alleles shorter than the wild-type *opa31* genotype except the *opa23* variant, which is also missing the intervening histidine. Thus, the *opa24–opa30* range, which would encompass variants that are one to seven residues shorter than wild-type, is a veritable “allelic barrens” for *D. melanogaster*.

### Embryonic failure is associated with rare Notch opa variants

**Figure 4:**
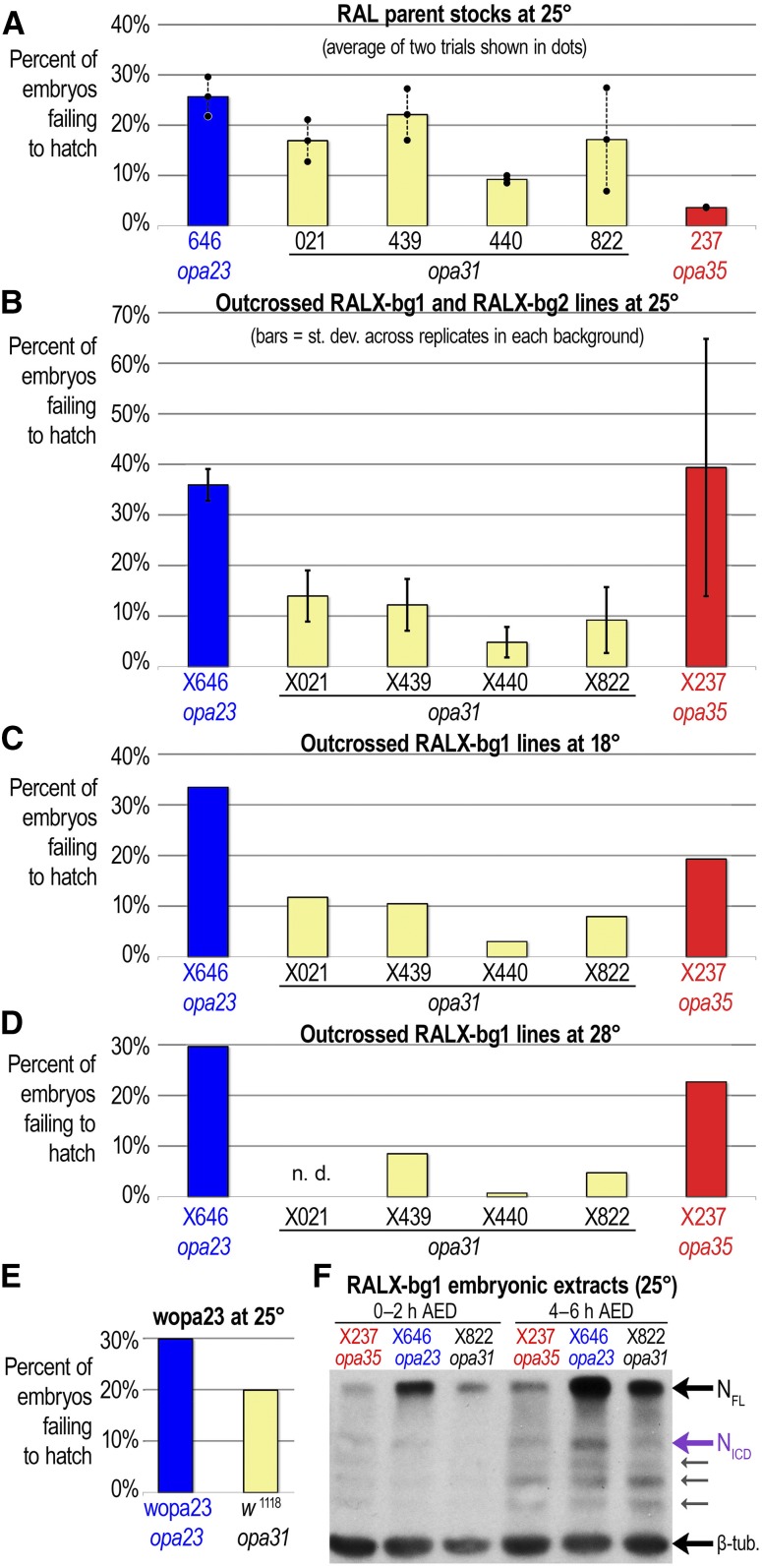
Lines with rare *opa* variants have aberrant Notch expression and suffer embryonic failure. (A) Among six isogenic RAL lines assayed for the rate of embryonic failure (failure to hatch as larva by all causes), RAL-646, which carries the short *opa23* allele, has the highest rate of failure, whereas RAL-237, which carries the long *opa35a1* allele, has the lowest rate of failure. Four different RAL lines carrying wild-type *opa31* alleles have intermediate levels of embryonic failure. Bars represent averages of two replicate trials shown as dots. (B–D) Embryonic failure is increased in outcrossed RALX lines carrying the extreme *opa* variants, whereas it is decreased in all of the *opa31* outcrossed lines at 25° (B), 18° (C), and 28° (D). Bars in (B) represent averages of four replicate trials, two from each background, except RALX021-bg1, which was conducted only once. Error bars represent ±1 SD. RALX237-bg1 and RALX237-bg2 have a consistently higher level of embryonic failure than all the outcrossed *opa31* lines, but the RALX237-bg2 failure rate is much higher than RALX237-bg1. (E) The recombined wopa23 line carrying a smaller region of the RAL-646 X chromosome and outcrossed into the *FM7* and *w*^1118^ background shows a higher level of embryonic failure than the *w*^1118^ stock. (F) Western blot of embryonic extracts from RALX-bg1 strains carrying different *Notch opa* variants from before (0–2 hr after egg deposition, or AED) and after Notch activation (4–6 hr AED), when the cleaved Notch intracellular domain (N_ICD_, purple arrow) is more prominent. Gels were probed with anti-NICD antibody and anti-β-tubulin antibodies. Smaller NICD degradation products can be seen below NICD (smaller gray arrows).

RAL-646 flies, which are homozygous for the short *opa23* rare variant allele, are much more difficult to expand than most other RAL stocks homozygous for the wild-type *opa31* allele. We attribute this in part to a much higher rate (27%) of embryonic failure in the RAL-646 stock relative to all five other RAL stocks that we tested at 25° (Figure 4A). Interestingly, RAL-237, which carries the long *opa35* rare variant allele, had the lowest embryonic lethality at 3.7% of all six lines tested. To further test that the two RAL stocks with rare *opa* variants have outlier embryonic failure phenotypes (very high in RAL-646 but very low in RAL-237), we also measured embryonic failure for the heterozygous RAL-142 stock (*opa31*/*opa32*) and the homozygous RAL-208 (*opa31*) and RAL-338 (*opa32*) stocks at room temperature (22–23°). We found that these had failure rates of 15.9%, 5.2%, and 5.2%, respectively, in the same range as all the *opa31* lines tested at 25°. Altogether, the seven RAL stocks that were homozygous or heterozygous for the two predominant alleles of *opa31* and *opa32* and raised at 22–25° had an average of 13.1% embryonic failure, which is less than half the failure seen in RAL-646.

To assess whether other issues distinguished the growth of these stocks, we measured fecundity at 25° (number of eggs laid in a 28-hr period, dusk-to-dusk). We found that the RAL-646 stock has a nearly average fecundity, demonstrating that this stock’s failure to thrive stems mainly from its increased embryonic failure, possibly caused by its *opa23 Notch* sequence. Of note, the inbred RAL-237 line again outperformed all other stocks in this regard (Table 2).

**Table 2:** RAL lines with the shortest and longest *opa* variants have average or above average fecundity

RAL Line	Genotype	Fecundity at 25°*^a^*	% of Maximum*^b^*
646	*opa23*	1436 eggs	63.1
021	*opa31*	1196 eggs	52.5
439	*opa31*	1699 eggs	74.6
440	*opa31*	742 eggs	32.6
822	*opa31*	1438 eggs	63.2
237	*opa35*	2276 eggs	100
AVG.		1465 eggs	64.3

a Number of eggs laid in 28-hr period beginning 2 hr before dusk until 2 hr after the next dusk.

b Average fecundity was 1465 eggs (64.3%) of maximum 2276 eggs by RAL-237.

To investigate whether the RAL-646 and RAL-237 lines were exhibiting outlier phenotypes in the embryonic lethality assays due to their variant X-linked *opa* repeats, we assayed this phenotype and others after introgressing the different RAL X chromosomes into two common backgrounds (see *Materials and Methods*). For background one (“bg1”), we used an *FM7c*/*N${}^{1}$* stock. We refer to this series of introgressed lines as the “RALX#-bg1” series in contrast to their “RAL-#” parent stocks. To rule out effects due to *N${}^{1}$* modifying suppressors in background one, we also created a series of introgressed RAL X lines using an *FM7a* stock (“bg2”). We refer to this second series of introgressed lines as the RALX#-bg2 series.

We found that embryonic failure increases significantly for both the *opa23* and the *opa35* outcrossed lines, whereas it becomes attenuated in eight different *opa31* introgression lines (compare A to B in Figure 4). While the *opa35* RAL-237 stock had the lowest embryonic failure in all experiments, the RALX237-bg2 has the highest embryonic failure at 60.6%. We found similar results when we repeated the embryonic failure assays at colder (Figure 4C, 18°) and warmer (Figure 4D, 28°) temperatures than 25°. Interestingly, these results suggest that the major allele *opa31* is optimal in outcrossed lines, whereas the *opa35* is optimal in an isogenized background with compatible suppressor modifiers. In contrast, the *opa23* allele is likely deleterious in most genomic backgrounds and at different temperatures, as we find that this continues to be the case when we recombine this region out of the X chromosome of RAL-646 (Figure 4E) in the wopa23 flies discussed later.

To determine whether the embryonic lethality differences in the *opa* allelic series is due to Notch proteostatic issues related to its pQ tract, we performed Western blot analyses comparing embryonic extracts from two time points corresponding to pre-Notch and post-Notch activation. Using an antibody specific to the Notch intracellular domain, NICD, we found that extracts from *opa23* embryos (RALX646-bg1) had substantially increased levels of full-length Notch receptor at both time points and higher levels of NICD at the postactivation time point, relative to extracts from the wild-type *opa31* embryos (RALX822-bg1) (Figure 4F). In contrast, extracts from *opa35* embryos (RALX237-bg1) had modestly lower levels of full-length Notch but oddly similar or slightly elevated levels of NICD relative to wild-type *opa31* embryos (Figure 4F). Thus, we found there are observable differences in the levels of both full-length Notch and NICD in the outcrossed lines carrying the most extreme *opa* variants, which may be related to their differences in embryonic survival.

### Bristle defects are associated with rare Notch opa variants

**Figure 5:**
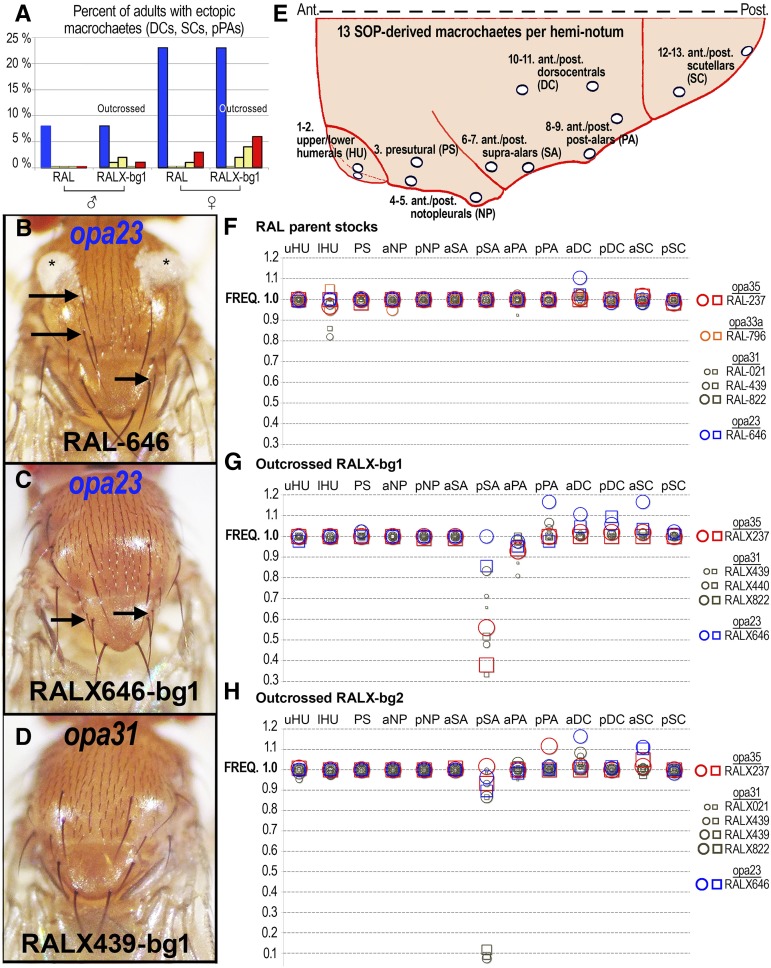
Rare *Notch opa* variants manifest macrochaete patterning defects. (A) Shown is a graph of the frequency of ectopic (*i.e.*, duplicated) bristles on the adult thorax (DCs, dorsocentral macrochaetes; SCs, scutellar macrochaetes; pPAs, posterior postalars) of male (left) and female (right) RAL stocks and outcrossed RALX-bg1 lines, carrying different *opa* variants at *Notch*. This defect is most pronounced for the RAL-646 and RALX646-bg1 lines (blue bars) carrying the rare *opa23* variant, but some intensification is also seen for the RALX237-bg1 line (red bars) carrying another rare variant, *opa35*. Lines carrying wild-type *opa31* alleles in either the parent RAL stocks or the RALX-bg1 derivative lines based on RAL-439, RAL-440, and RAL-822 (yellow bars) rarely have ectopic bristles. (B–D) Shown are representative adult nota with ectopic bristles (arrows) in the parent RAL-646 (B) and the outcrossed RALX646-bg1 line (C), but not the RALX439-bg1 line (D). Cuticle fluff (asterisks in B) is frequently seen in the RAL-646 flies. (E) Cartoon of hemi-notum and its 13 macrochaetes, abbreviated as follows: (1–2) upper and lower humerals (uHU, lHU); (3) presutural (PS); (4–5) anterior and posterior notopleurals (aNP, pNP); (6–7) anterior and posterior supra-alars (aSA, pSP); (8–9) anterior and posterior postalars (aPA, pPA); (10–11) anterior and posterior dorsocentrars (aDC, pDC); and (12–13) anterior and posterior scutellars (aSC, pSC). Anterior is to the left. Posterior is to the right. Top of image is the dorsal midline. (F) Frequency (FREQ) of macrochaete occurrence in parent RAL stocks (different colored shapes) for female (circles) and male (squares) flies. (G) Frequency of macrochaete occurrence in 5× outcrossed RALX stocks using *FM7c* males (bg1). Outcrossed lines are homozygous for the RAL X. The failure to form pSA is also seen in the *FM7* balancer lines (data not shown). (H) Frequency of macrochaete occurrence in 5× outcrossed RALX stocks using *FM7a* males (bg2). Outcrossed lines are homozygous for the RAL X. These data show that the RALX646 (blue *opa23*) and RALX237 (red *opa35*) outcrossed lines produce ectopic posterior postalar, dorsocentral, and scutellar macrochaetes.

To determine the extent to which classic *Notch* phenotypes are associated with RALX outcrossed lines carrying rare *opa* variants, we measured the frequency of duplicated macrochaetes on the notum of adult flies in both parent and the RALX introgressed lines carrying variant *Notch opa* alleles. First, we scored the frequency of ectopic dorsocentral (DC), scutellar (SC), and posterior postalar (pPA) macrochaete-type bristles (Figure 5, A–D). We found that the RAL-646 (*opa23*) parent line has a high rate of ectopic macrochaetes in both males (8%) and females (23%) relative to representative controls. This effect was seen to a lesser degree in the parent RAL-237 stock, which carries the *opa35* variant of *Notch*. In the outcrossed lines, we found that the *opa23* line, RALX646-bg1, maintains the same high rate of ectopic macrochaetes similar to the parent RAL-646 stock, whereas the *opa35* line, RALX237-bg1, exhibits an increased rate of ectopic macrochaetes that is higher than both its parent RAL stock and all of the outcrossed lines carrying wild-type *Notch opa* repeats (Figure 5A). In both of the two variant outcrossed lines, the highest levels of ectopic bristles occur in females, more than three-times higher than outcrossed males, suggesting that this phenotype may be sensitive to imprecision in dosage compensation.

To understand whether specific macrochaete bristles are affected by the various *Notch opa* alleles, we scored the relative presence and absence of each of the 13 macrochaete bristles of the adult hemi-notum in our RAL parent lines and outcrossed RALX-bg1 and RALX-bg2 lines (Figure 5, E–H). These results show that the posterior PA, anterior and posterior DC, and the anterior SC bristles are ectopically produced in lines carrying rare, variant *Notch opa* alleles. We note a general failure to form the posterior supra-alar (pSA) macrochaete in most outcrossed RALX-bg lines regardless of *Notch opa* status. Because we see a similar effect in *FM7* flies, which we used as the background for introgressing the RAL X chromosomes, we attribute this particular bristle defect to autosomal modifiers carried in the *FM7* genetic background that likely affect the specific prepatterning signals driving proneural cluster expression at this bristle position.

### Does a rare Notch opa variant suppress a key scute inversion?

Balancers are chromosomal tools that have been used in hundreds of genetic studies in *Drosophila*, including much of the work involving *Notch* (Lindsley and Grell 1972; Ashburner 1989). In the process of genotyping the *N*^1^ and *N^TA17V^* stocks, which are kept over the multiply-inverted *FM7c* X balancer chromosome, we discovered that this balancer carries a rare *opa33b* allele. After sequencing many other X balancers, we found that most carry the rare *opa33b* allele while some carry *opa32* (Figure 2). Thus, we found that no classical X balancer chromosome carries the major *opa31* allele. This includes *FM1*, *FM4*, *FM6*, *FM7a*, *FM7c*, and *Basc*. In contrast, this *Notch opa33b* variant is rare in the RAL stocks (2/41 RAL lines genotyped, see Figure 2).

To understand the association of *Notch opa33b* with most balancers, we first noted that all such X balancer chromosomes (*FM1*, *FM7a*, *FM7c*, and *Basc*) are linked to the *white apricot* allele *w^a^*, whereas those carrying *Notch opa32* (*FM4* and *FM6*) are linked to the wild-type *white* locus. (The latter two balancers likely swapped the *white–Notch* region during recombination events associated with creation of these hybrid X balancers.) In approximately 1929, Sidorov subjected *w^a^* flies to X-ray mutagenesis and generated the *scute* inversion allele *In(1)sc${}^{8}$* (Sidorov 1931). The *In(1)sc${}^{8}$* inversion was one of the founding inversions used in the “First Multiple” inversion balancers. Thus, we sequenced both *In(1)sc${}^{8}$* and the *w^a^* stock (BDGP #148) and found both carry *opa33b* (Figure 2). To confirm the simple conclusion that the balancer *Notch opa* variant originated with the *w^a^* stock, we genotyped the related *sc*^7^ inversion, *In(1)sc*^7^, which was independently produced by mutagenesis of *w^a^*, and found that it also carries *opa33b* (Figure 2).

**Figure 6:**
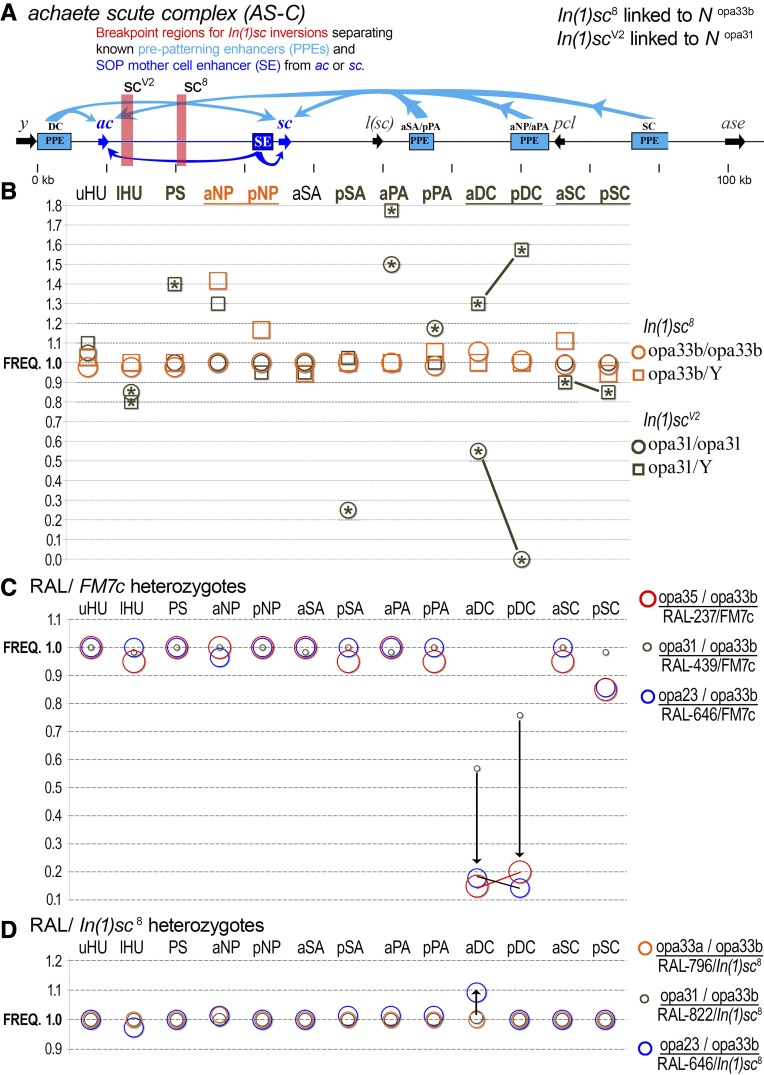
A *scute* inversion carrying the wild-type *opa31 Notch* allele manifests a severe bristle patterning defect that is not seen with an equivalent *scute* inversion carrying the rare expanded *opa33b* allele. (A) The *AS-C* locus and positions of the *sc^V^*^2^ (linked to *opa31*) and *sc*^8^ (linked to *opa33b*) inversion breakpoints (light red vertical bars). Also shown are the diverse prepatterning enhancers (blue PPE boxes), which normally drive both *ac* and *sc* (blue arrows) in different proneural clusters of developing wing imaginal discs. In addition, the SOP mother cell enhancer (dark blue SE box), which drives both *ac* and *sc*, is also shown. Although each inversion is linked to a different *Notch opa* allele carried within the inversions, both inversions separate the same set of known enhancers. The DC PPE also coincides with the *yar* noncoding transcript (Soshnev *et al.* 2011). (B) A comparison of macrochaete patterning defects shows that *sc^V^*^2^ has more deleterious effects than *sc*^8^, consistent with a suppressor role for *opa33b* expanded *Notch*. (C) A comparison of macrochaete defects for RAL F1 heterozygotes over *FM7c*, which carries the *sc*^8^ inversion allele, shows that heterozygotes with the short and long variants are severely deficient in specifying DC and pSC bristles relative to a representative wild type heterozygote. (D) RAL stocks over the *sc*^8^ inversion allele by itself (\emph{i.e.}, not in a complex balancer chromosome), shows more modest effects compared to the *FM7c* heterozygotes and further suggests the presence of *N*^*1*^ modifiers in the latter (see text).

To determine whether the *opa33b* variant of *Notch* suppresses or enhances the *sc*^8^ phenotype caused by a separation of diverse prepatterning enhancers and the SOP mother cell enhancer from *achaete* or *scute*, we identified an *sc* inversion allele that has the closest known breakpoint to the *sc*^8^ breakpoint, *sc^V^*^2^ (Figure 6A). This *sc^V^*^2^ allele’s breakpoint is 6 kb upstream of the *sc*^8^ breakpoint and separates the same set of known prepatterning and SOP enhancers present throughout the ∼105-kb-long *AS-C* (Figure 6A). We genotyped this inversion allele and found that it encodes a wild-type *opa31 Notch* allele (see Figure 2). Thus, in the absence of additional modifiers and suppressors, differences in the bristle patterning defects of the *scute* alleles *sc^V^*^2^ and *sc*^8^ constitute another test of the hypothesis that the precise *Notch opa* length is a functional modifier of Notch-Delta network signaling behavior as suggested by the severe shape of the allelic distribution (Figure 2) and both the embryonic lethality (Figure 4) and bristle defects (Figure 5) associated with introgressed RAL X chromosomes carrying rare *opa* variants.

We found that the *sc^V^*^2^ line, which is linked to *Notch opa31*, has a more severe and penetrant bristle phenotype compared to the *sc*^8^ inversion, which is linked to *Notch opa33b* (Figure 6B). For example, *sc^V^*^2^ males have extremely high rates of ectopic DCs while *sc^V^*^2^ females have high rates of DC patterning defects. Empty sockets are not observed in females, suggesting that the defect is in underspecification of sensory organ precursors in females and overspecification in males. In stark contrast, *sc*^8^ males and females have almost normal DC bristle patterning. This result is consistent with a role for the *opa33b* allele of *Notch* in partially suppressing *AS-C* insufficiency caused by the inversion’s localization of enhancers away from *ac* or *sc*.

We also conducted two related experiments that compare the bristle phenotypes in heterozygous females carrying either the *In(1)sc*^8^ or *FM7c* balancers and RAL X chromosomes carrying different *opa* variants. Genome-wide modifiers of *Notch* variants, including other pQ-variable loci, may have been acquired in stocks carrying balanced *Notch* mutants. Thus, we crossed our RALX-bg1 lines to *FM7c*/*N*^1^ flies and scored the heterozygous balancer females (RALX/*FM7c*). We found that the *opa23* and *opa35* heterozygotes are similarly severely deficient in the formation of DC and posterior SC macrochaetes relative to *opa31* heterozygotes (Figure 6C). At several other bristle positions, the *opa35* (lHU, pSA, pPA, aSC) and *opa23* (aNP) variants are modestly affected relative to *opa31* heterozygotes. For comparison we conducted similar experiments with RALX/*In(1)sc*^8^ heterozygotes, which would be deficient in the postulated suppressors of the *Notch${}^{1}$* phenotype. We found that the *opa23* heterozygotes do not show a severe bristle phenotype in this “naive” background. This suggests to us that the *N*^1^/*FM7c* balanced stock has likely acquired suppressor modifiers of the *N*^1^ phenotype.

We hypothesized that some classic *Notch* mutants may have acquired suppressors via changes in the *opa* repeats after decades of maintenance at stock centers. The slightly expanded *opa32* version of *Notch* was previously reported for the classic alleles *N^Co^* and *N*^60^*^g^*^11^, but this extra glutamine is thought to be phenotypically silent (Kidd *et al.* 1986; Lyman and Young 1993). To determine whether *Notch* opa repeats are coevolving with the primary mutations of *Notch* mutant alleles, we genotyped several additional classical *Notch* mutant stocks. Remarkably, our survey of *N*^1^, *N^ts^*^1^, *N^ts^*^2^, *N^nd^*^0^, *N^TA^*^17^*^V^*, and *N^Ax-ts^*^1^ did not turn up a single length variant different from *opa31* (Figure 2 and Figure 3) despite the number of *opa* variants we had found by surveying RAL stocks. This result suggests to us that perhaps changes in the *opa* repeat number by contraction or expansion are generally deleterious in combination with other lesions at this locus.

### The Notch opa23 locus gives bristle defects and notched wings

**Figure 7:**
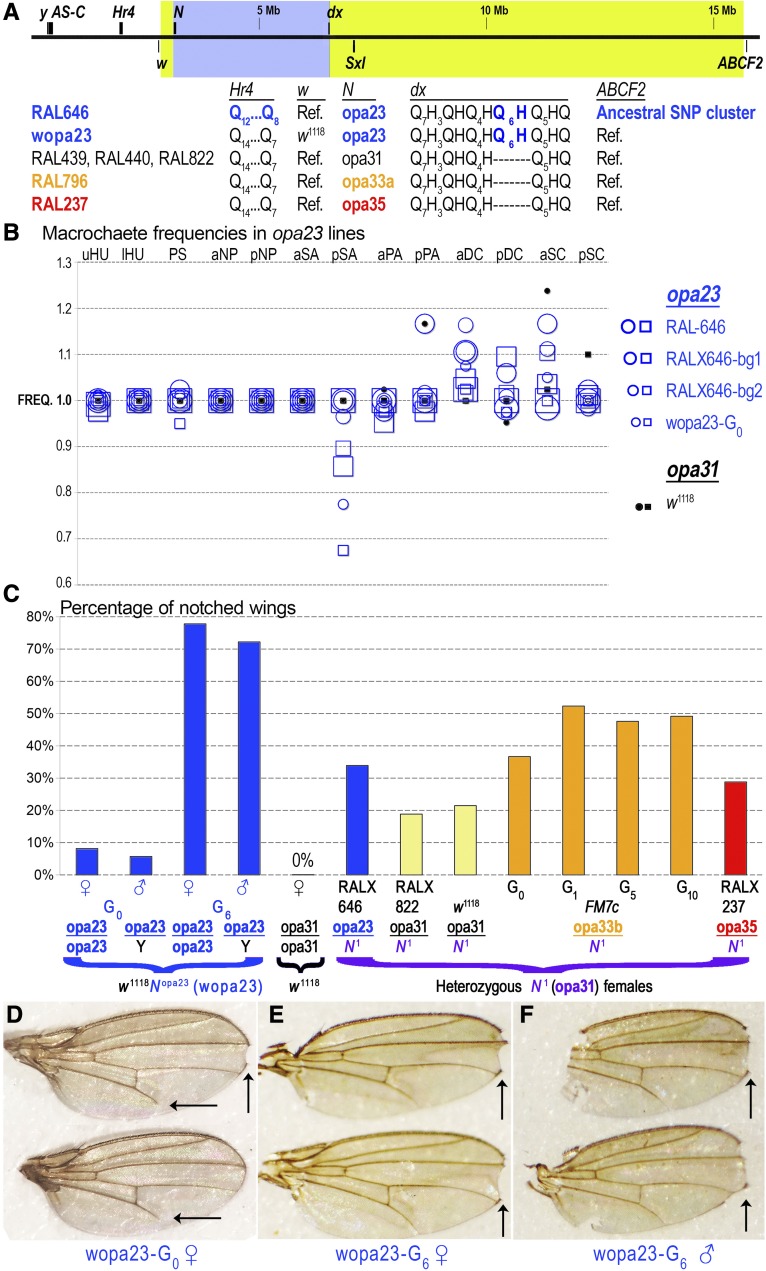
The X chromosomal region carrying the rare *Notch opa23* variant is associated with classic Notch phenotypes. (A) Shown is the region from chromosome X containing *Notch*. These genes include: *yellow* (*y*), the *achaete scute* complex (*AS-C*), *Hr4*, *white* (*w*), *Notch* (*N*), *Deltex* (*dx*), *Sex lethal* (*Sxl*), and *ABCF2* (*CG9281*). Below the chromosomal graphic are the various polymorphisms distinguishing RAL-646 from other RAL stocks or RAL-646-derivative lines, including the wopa23 line in which we introgressed the region of the *Notch* locus (blue shaded area) and some of the nearby flanking regions (exact breakpoints of fragment lie within the yellow–green shaded areas) into control non-RAL backgrounds. (B) Shown are the macrochaete frequencies for RAL-646, RALX646-bg, and wopa23 line in the same convention as previous figures. (C) Shown is a graph depicting the frequency of wing notching in various lines. The original wopa23 generation (wopa23-G_0_) produced notched and/or nicked wings in males and females at a modest but not insignificant rate. However, following six generations of selection for notched wing flies (wopa23-G_6_), this rate increased dramatically to 72% in males and 78% in females. In contrast, selection for notched wings in the *N*^1^/*FM7c* stock failed to increase the rate of notching past 53% even after 10 generations of selection. (D) Shown are a pair of wings from a *wopa23*-G_0_ female prior to selection for notched wings. Vertical arrow shows a classic wing notch. Horizontal arrows in each wing show an additional defect of incomplete L5 wing vein formation, albeit at a lower frequency than notched wings. (E, F) Wings from female (E) and male (F) wopa23 flies after six generations of propagating only flies with notched wings. These flies have more deeply serrated notches than the unselected wopa23 stock, but the incomplete L5 wing vein phenotype disappears.

To rule out phenotypic effects caused by X-linked loci other than *Notch*, we recombined the rare and interesting *Notch opa23* allele out of its RAL-646 X chromosomal background (see *Materials and Methods*). We used the parent RAL-646 stock, *w*^1118^ fly stock and *FM7* balancer stocks to produce a white-eyed “wopa23” line, carrying a much smaller portion of the RAL-646 X chromosome spanning from *Notch* to some crossover point between *dx* and *ABCF2* (*CG9281*) (Figure 7A). We scored macrochaete phenotypes for the wopa23 flies and compared them to our series of outcrossed *opa23* lines (Figure 7B). This showed that male wopa23 flies have missing presutural macrochaetes approximately 5% of the time, which is a defect not seen in RAL-646, RALX646-bg1, RALX646-bg2, or *w*^1118^ flies of either sex. Elevated levels of ectopic aDC bristles are seen in the wopa23 flies, but these are comparable to the *opa23* parent and outcrossed lines. We also found that our wopa23 male and female flies displayed the eponymous *Notch* phenotype of notched and nicked wing tips (Figure 7, C–F, and vertical arrows). This suggests that there were X-linked suppressors of *Notch opa23*, which were recombined away in this line. A smaller number of flies had a wing vein patterning defect in which the L5 vein fails to reach the wing margin (horizontal arrows in Figure 7D).

To understand the notched wing phenotype of the wopa23 flies better, we put various RALX-bg1 X chromosomes over an X chromosome carrying the dominant *N${}^{1}$* allele, which produces notched wings at a certain frequency. We then scored the frequencies of notched and/or nicked wing tips in the F_1_ heterozygous nonbalancer females. Our *N*^1^ cross results show that the *opa23* and *opa35* alleles have the smallest suppression effect on notched wings in heterozygous F_1_ females. This suggests that both contractions and expansions of the wild-type *opa31* repeat length are types of lesions at the *Notch* locus.

We also performed an experiment on the *N*^1^/*FM7c* and wopa23 flies in which we selected for notched wings over several generations. While we increased the number of notched wings in *N*^1^ females modestly from 34 to 50% after one generation of selection (*N*^1^ is lethal in males), we were unable to achieve further increases even after 10 generations of selection (Figure 7C). In contrast, we substantially increased the rate of notched wings in both males and females from <10% to >70% in our wopa23 stock after only a few generations of propagating only flies with notched wings (Figure 7C). These “Super-Notch” wopa23-G_6_ flies lose the L5 wing vein phenotype that we first saw with the wopa23-G_0_ generation, even while the severity of wing notching was increasing (compare Figure 7D to Figure 7, E and F). We also regenotyped the Kodani inversion in the wopa23-G_0_ and Super-Notch wopa23-G_6_ lines and found that both still carried at least one copy of the *In(3R)K* inversion despite the three generations of outcrossing involved in the recombination screen (File S1).

## Discussion

We discovered a plethora of functional *Notch opa* variants by sampling the Raleigh lines (DGRP) and genotyping multiple independent clones from each line by Sanger sequencing. We found that almost half of the RAL haplotypes (19/41) do not carry the standard *opa31* variant and are not being accurately genotyped by high-throughput sequencing and assembly. Importantly, alleles characterized by extremely short or long *opa* repeats have common developmental defects, including classic *Notch* phenotypes affecting macrochaete patterning and wing notching. These phenotypes intensify when their X chromosomes or regions of the X chromosome containing *Notch* are outcrossed and/or recombined out into other backgrounds. The outcrossing effect suggests that these particular isogenic RAL lines carry both linked and unlinked suppressor modifiers. It will be interesting to determine the extent to which the 205 DGRP isofemale lines, established after 20 generations of inbreeding initially 1500 Raleigh females, are enriched for compatible alleles (David *et al.* 2005).

The Line-646 DGRP mis-assembly at *Notch* is an example of the problematic nature of genotyping the *opa* sequences and of the repeat assembly problem in general. This problem is exacerbated by the need for flanking nonrepetitive “anchor” sequence to accurately start or end the repeat alignments in the correct position, as this further reduces the remaining read length that can span into the repeats. For the *Notch opa* repeats, a single 75 nucleotide (nt) read cannot span the wild-type length of 93 bp even without anchor sequence. From what we have seen, 91% of *opa* length variants (10 of 11 observed variant length classes) have a greater number of repeats than wild-type. If a minimum of two flanking non-CAX codons are used for anchoring sequence, this leaves only 23 repeat CAX codons in a 75-nt read (6 + 69). Thus, even the shorter RAL-646 *opa23* repeats cannot be spanned by a single 75-nt read. In summary, we found that the important *Notch opa* repeats are best sequenced and assembled using sequence reads >140 bp long (3 bp × 40 triplet repeats + 10 bp × 2 anchor sequences).

Tautz reported hypervariability for the *Notch opa* repeats of *D. melanogaster* after seeing four length variants from 11 independent isogenic female lines established from North and South America, Europe, Asia, and Australia (Tautz 1989). These four alleles encode the pQ range of Q_13_HQ_17-20_ and most commonly correspond to wild-type *opa31* and *opa32* (81.8%). Two rare variant haplotypes corresponded to an *opa33c* encoding Q_13_HQ_19_, and an *opa34b* encoding Q_13_HQ_20_ (Figure 2). If we consider just the three North American isofemale lines genotyped by Tautz, then these give a 2:1 ratio of *opa31* to *opa32*, which very roughly approximates the 3:1 ratio we obtained from genotyping the isofemale Raleigh lines of the DGRP (Table 3). However, this ratio falls to 0.8:1 when all the worldwide isofemale lines are considered. The single isofemale line from Japan was homozygous for *opa34b*, whereas the single isofemale line from the USSR was heterozygous for *opa33c*/*opa31*. Both of these variants have not yet been genotyped by us in the resequenced DGRP lines. Thus, it will be interesting to see if different *opa* alleles are found in different world populations.

**Table 3:** Comparison with worldwide distribution of *Notch opa* repeat variants

Study	Source	No. of Lines*^a^*	*opa31*	*opa32*	*opa33*	*opa34*	All Other *opa* Length Variants
Rice *et al.* (this study)	North America	41	54.9%	18.3%	12.2%	2.4%	11.9%
Tautz (1989) subset*^b^*	North America	3	66.7%	33.3%	0.0%	0.0%	0.0%
Tautz (1989) all	Worldwide	11	36.4%	45.4%	4.5%	9.1%	0.0%

a Number of iso-female lines genotyped.

b 3/11 iso-female lines from worldwide populations (Tautz 1989).

Among the new *opa* variants, we discovered the rare short *opa23* allele that causes embryonic lethality in many backgrounds. Of all the rare *opa* alleles that we discovered by sampling DGRP RAL lines and other stocks, the *opa23* allele has the greatest length change relative to the wild-type *opa31*. It is also the only *opa* allele not encoding the histidine residue internal to the pQ tract. Both the parent RAL-646 and RALX646 introgressed lines, bg1 and bg2, correspond to the stocks and lines with the greatest bristle patterning defects, a classic *Notch* phenotype. This chromosomal region around the *Notch* locus is also responsible for producing notched wings when recombined into different backgrounds, presumably because it loses X-linked suppressor modifiers of *Notch opa23*.

The distribution of *opa* length variants suggests extreme purifying selection against the shorter would-be alleles in the barrens range (*opa* 24–30), and somewhat more moderate purifying selection against the many different longer variants in the *opa* allelic jungle range (*opa* 31–38). Given the *Notch*-like phenotypes associated with extreme variants in the skewed distribution of *opa* genotypes (Figure 2), the allelic barrens is unlikely to be an artifact of poor sampling. We propose that the existence of *opa23* is supportive of the reality of the barrens because it is missing the codon for histidine. On the far short side of the barrens, *opa* alleles may become semiviable provided the interrupting histidine is missing. In the presence of an intervening histidine residue, the *opa*-encoded pQ peptide may behave differently, as seen in other contexts (Sharma *et al.* 1999; Sen *et al.* 2003; Kim 2013). In this regard it is interesting that the longest *opa* variant we have observed also has a contiguous stretch of 23 Qs. Thus, 23 Qs are the longest uninterrupted tracts we have observed in either short or long *opa* variants. Why this should be so must be specific to the roles played by NICD.

Perutz explained the 40 glutamine pathological threshold via the amyloid structure that is able to form from two stacked 20-residue-long rings (Perutz *et al.* 2002). In this cross-β amyloid nanotube, each glutamine side group forms a hydrogen bond with another side chain in the adjacent ring. These side-chain hydrogen bonds are in addition to the ones along the peptide backbone, typical of all β-sheet structures. However, the observed *opa* repeat distribution also suggests possible lethality of expansions past Q_23_. Because this is significantly below the pathological limit, we think either of two possible explanations will eventually be found to apply. First, a long pQ tract with only a single intervening histidine might behave similarly to an uninterrupted tract in some respects, such that a new Perutz amyloid threshold exists near 38–40 residues. Second, some other nonamyloid structure, possibly an interdigitated β-sheet interaction between NICD and one or more other interacting proteins, exists. These interactions may be too strong when the tract is abnormally expanded. Similarly, the allelic barrens might represent the lethality of contracting the pQ tracts below Q_13_ (left-side) or Q_17_ (right-side) if doing so impacts specific critical interactions.

We find only three synonymous A/G (puRine) substitutions in all of the known *opa* sequences (Figure 3). These three synonymous polymorphisms occur at specific positions within and near the long (CAG)_7_ triplet repeats (an unspecified number of the 11 Tautz sequences have the *2 polymorphism as it occurs in that consensus). The location of these synonymous substitutions is consistent with two empirical observations. First, indel mutations scale as a function of repeat number for diverse repeat unit lengths and sequences (Ananda *et al.* 2013). Second, nucleotide substitution rates tend to be elevated around indel polymorphisms (Ellegren 2000; Schlötterer 2000). At a superficial glance, the occurrence of these substitutions across the diversity of *opa* genotypes (Figure 3) suggests they are independently recurrent mutations. Alternatively, *opa* gene conversion events may be common at this locus, in which case it would be difficult to produce simple relationships between these alleles.

Why do we find rare variant alleles like *N^opa23^* if they are deleterious in many backgrounds? Some of the microchaete defective alleles of *Notch* (*N^Mcd^*) encode C-terminal truncations of NICD that still have the N-terminal ankyrin repeats as well as both nuclear localization signals but are missing the *opa*-encoded pQ tract (see Figure 1A) (Ramain *et al.* 2001). Such *N^Mcd^* alleles are pupal lethal, suggesting an important role for the disordered C-terminal half of NICD and its pQ tract. This is consistent with our results, which suggest that contractions and deletions that reduce or eliminate the *opa* repeats are also lethal. Thus, one possible answer is that variant *Notch opa* alleles are continuously being generated *de novo* because of intrinsic instability associated with repeat sequences in general (Ananda *et al.* 2013). These variants are likely subject to intense selection because there are at least three different ways in which *opa* repeat variability can affect Notch function. First, *opa* repeat variability may affect the absolute levels of Notch protein due to increased degradation during cotranslational membrane targeting, processing, or in the mature full-length membrane bound or cleaved NICD forms (Figure 4D). Second, *opa* repeat variability may affect the strength of interactions NICD makes with other cofactors in the nucleus as discussed above. Indeed, the Notch coactivator Mastermind, which is recruited by NICD and required for its activity (Wu *et al.* 2000; Fryer *et al.* 2002, 2004; Kovall 2007), has several pQ tracts that are under substantial selective constraint (Newfeld *et al.* 1991, 1993, 1994). Third, *opa* repeat variability may affect the ability of NICD to assume the distinct conformational shapes that allow pQ-dependent self-association or interaction with the Su(H) transcription factor (Kelly *et al.* 2007). In summary, the distribution of *opa* length variants of *Notch* likely represents a snapshot summary of the ongoing selection acting on these unstable but functionally necessary *opa* repeats and the precise *opa* repeat number.

Interestingly, we found that almost twice (1.9×) as many of the sampled radiation-resistant RAL lines (Vaisnav *et al.* 2014) encoded non-*opa31 Notch* variants compared to the sampled radiation-sensitive RAL lines (Table 1). This suggests to us that these lines may feature proteostatic stress in the endoplasmic reticulum (ER), where the Notch membrane receptor is cotranslated with membrane integration (Fortini 2009; Yamamoto *et al.* 2010). Similarly, upregulation of the unfolded protein response (UPR), which is induced by misfolded proteins in the ER, is known to confer treatment resistance to some cancers (Nagelkerke *et al.* 2014). Enrichment for Notch pQ variants in radiation-resistant lines also suggests that *Notch* is one of only a few such loci that are both variable and capable of causing systemic stress. If so, then it will be interesting to identify the mechanisms for radiation-resistance in the few such *opa31* lines that we have genotyped.

We conclude by discussing whether there is a role for a tunable *Notch* locus in the context of developmental canalization, particularly in light of distinct bristle patterning phenotypes by equivalent *scute* inversions carrying different *Notch opa* variants (Figure 6). The induction of proneural clusters for specific macrochaetes involves distinct pathways targeting a number of independent “prepatterning” enhancers (PPEs) at the *achaete scute* complex (*AS-C*) (Martínez and Modolell 1991; Skeath *et al.* 1992; Gómez-Skarmeta *et al.* 1995; Modolell and Campuzano 1998). Importantly, mutations in genes for Notch-Delta signaling components can affect specific bristles, and indeed sensory bristle-specific regulatory modules and the *AS-C* gene copy number are dynamically evolving (Galant *et al.* 1998; Negre and Simpson 2009). In addition, proneural cells transition from experiencing diverse prepatterning signals at different presumptive bristle positions to the same lateral inhibition regulatory network featuring Notch-Delta signaling. As the 26 symmetric macrochaete sensory organs on the adult notum are also monomorphic traits, these circuits must also function in the presence and absence of male dosage compensation of X-linked genes, including the *AS-C* and *Notch* genes. Thus, Notch-Delta signaling must become highly canalized to build the stereotypical macrochaete-type sensory organ at several positions receiving distinct signaling cues and operating at slightly different dosage levels. Some preliminary work has already been done to model parameter space across different mechanistic contexts (*e.g.*, lateral inhibition, boundary formation, asymmetric cell fate specification) (Matsuno *et al.* 2003; Barad *et al.* 2010, 2011; Sprinzak *et al.* 2011; Shaya and Sprinzak 2011; Shimizu *et al.* 2014). In this context, it will be interesting to determine in future studies whether there are functional associations between distinct *Notch opa variants* and other pQ-encoding loci throughout the genome. If this is found to be the case, then the *Notch* locus could harbor an important evolvable repeat variable that influences the signaling characteristics of Notch-Delta regulated circuits.

## 

## Supplementary Material

Supporting Information
