# Supplementary material for: The Nature, Extent, and Consequences of Genetic Variation in the opa Repeats of Notch in Drosophila
Source: G3 (Bethesda). 2015 Sep 10;5(11):2405–19. doi: 10.1534/g3.115.021659 (PMC4632060; doi:10.1534/g3.115.021659)
Supplement: Supporting Information [file supp_5_11_2405__index.html]

The Nature, Extent, and Consequences of Genetic Variation in the opa Repeats of Notch in Drosophila — Supporting Information 

# The Nature, Extent, and Consequences of Genetic Variation in the *opa* Repeats of *Notch* in *Drosophila*

## Supporting Information for Rice *et al.*, 2015

**Files in this Data Supplement:**

- Supporting Information - Files S1-S3 (PDF, 662 KB)
- File S1 - Figure showing genotyping results for the Kodani inversion. (PDF, 150 KB)
- File S2 - Deconstruction of the original DGRP-646 ("Line646") sequencing reads and alignment. (PDF, 296 KB)
- File S3 - Figure showing the separate embryonic assay results for the RALX-bg1 and RALX-bg2 series. (PDF, 155 KB)
