# Supplementary figures and images for: The Nature, Extent, and Consequences of Genetic Variation in the opa Repeats of Notch in Drosophila
Source: G3 (Bethesda). 2015 Sep 10;5(11):2405–19. doi: 10.1534/g3.115.021659 (PMC4632060; doi:10.1534/g3.115.021659)

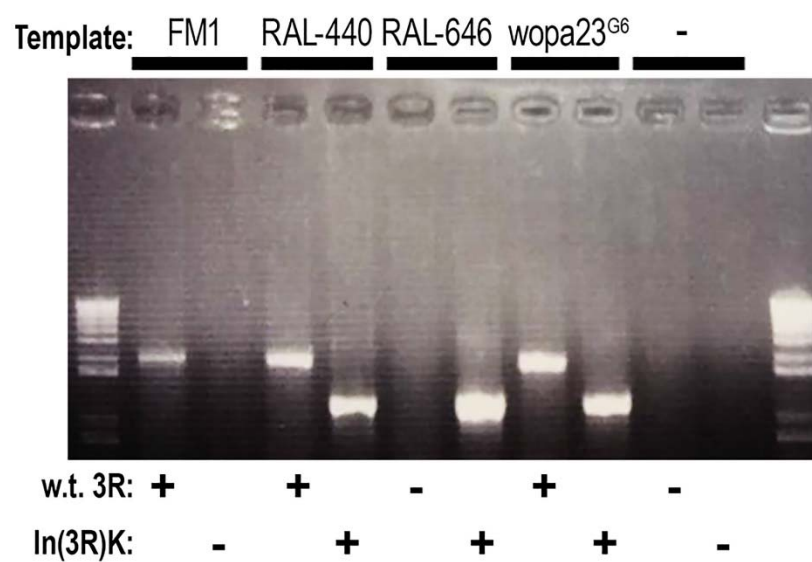

**File S1** Figure showing genotyping results for the Kodani inversion.

Supplement: Supporting Information [file supp_g3.115.021659_FileS1.pdf]
