# Supplementary material for: The Nature, Extent, and Consequences of Genetic Variation in the opa Repeats of Notch in Drosophila
Source: G3 (Bethesda). 2015 Sep 10;5(11):2405–19. doi: 10.1534/g3.115.021659 (PMC4632060; doi:10.1534/g3.115.021659)
Supplement: Supporting Information [file supp_g3.115.021659_FileS2.pdf]

Rice et al. (2015)

Supplementary File 2. Deconstruction of the original DGRP-646 (“Line646”) sequencing reads and alignment

1. We retrieved all original Illumina sequencing reads matching the exact CDS for the start (“PQ_9...”) or end (either “...Q_9L” or “...Q_5LGGLE”) of the Notch “opa” repeats underlying the “Line646” assembly (SRR835083). After confirming that the sequencing reads mapped to Notch CDS, we aligned and trimmed the sequences to focus on the opa repeat regions as shown below.

2. Aligning to reference (asterisked), we count a total of 67 mismatches from eight mismatched reads (10/13 reads). Furthermore, 3 of 4 codons spanning 5′-CAT (His) encode 5′-CAR (Gln). Number of mismatches (highlighted in fuchsia) relative to the reference sequence are given. Dashes indicate the region deleted in the inferred true alignment (explained in step #4 below).

```
REF.        *  CCCCAGCAACAGCAGCAGCAGCAGCAACAGCAACAGCAGCAACATCAGCAGCAGCAACAGCAGCAGCAACAGCAGCAGCAGCAACAGCAGCAGCAACTC
2766069     1  CCCCAGCAACAGCAGCAGCAGCAGCAGCAGCA----------------------
2766082     2  CCCCAGCAACAGCAGCAGCAGCAGCAGCAGCAACAGCAGCAGC-----------
2766083     6  CCCCAGCAACAGCAGCAGCAGCAGCAGCAGCAACAGCAGCAGCAAAAGCAGCAGCAGCAACA
2766075    25                           CCACAAAAAGCACAAGAACAAGACACCGACAACACCACCAGCAACAGCAGCAGCAGCAACAGCAGCAGCAACTC
2766076     9                                ---------CAACACCCCCCCGCGAAACAGCAGCAGCAACAGCAACAGCAGCAACAGCAGCAGCAACTC
2766089     2                                -----------GCAGCAGCAGCAGCAACAGCAGCAGCAACAGCAGCAGCAGCAACAGCAGCAGCAACTC
2766072    10                                -----------GCAGCAGCAACGCCACCGGCACCACCAACACCAGCAGCAGCAACAGCAGCAGCAACTC
2766087     1                                --------------GCAGCAGCAGCAACAGCAGCAGCAACAGCAGCAGCAGCAACAGCAGCAGCAACTC
2766074    10                                ---------------CCCCCACCACAACAGCACCACAACCAGCAGCAGCAGCAACAGCAGCAGCAACTC
2766092     0                                                                GCAGCAACAGCAGCAGCAGCAACAGCAGCAGCAACTC
2766078     1                                                                       CCGCAGCAGCAGCAACAGCAGCAGCAACTC
2766088     0                                                                         GCAGCAGCAGCAACAGCAGCAGCAACTC
2766090     0                                                                             CAGCAGCAACAGCAGCAGCAACTC
```

3. Removing reads with >2 mismatches in the opa repeat region reveals the origin of the DGRP “Line646” consensus of a single non-synonymous substitution (H→Q), two additional synonymous substitutions, and no indels. Three of the four mismatches are present in at least three sequences, suggesting that these are polymorphisms.

```
REF.        *  CCCCAGCAACAGCAGCAGCAGCAGCAACAGCAACAGCAGCAACATCAGCAGCAGCAACAGCAGCAGCAACAGCAGCAGCAGCAACAGCAGCAGCAACTC
2766069     1  CCCCAGCAACAGCAGCAGCAGCAGCAGCAGCA----------------------
2766082     2  CCCCAGCAACAGCAGCAGCAGCAGCAGCAGCAACAGCAGCAGC-----------
2766089     2                                -----------GCAGCAGCAGCAGCAACAGCAGCAGCAACAGCAGCAGCAGCAACAGCAGCAGCAACTC
2766087     1                                --------------GCAGCAGCAGCAACAGCAGCAGCAACAGCAGCAGCAGCAACAGCAGCAGCAACTC
2766092     0                                                                GCAGCAACAGCAGCAGCAGCAACAGCAGCAGCAACTC
2766078     1                                                                       CCGCAGCAGCAGCAACAGCAGCAGCAACTC
2766088     0                                                                         GCAGCAGCAGCAACAGCAGCAGCAACTC
2766090     0                                                                             CAGCAGCAACAGCAGCAGCAACTC
“Line646”   3  CCCCAGCAACAGCAGCAGCAGCAGCAGCAGCAACAGCAGCAGCAGCAGCAGCAGCAACAGCAGCAGCAACAGCAGCAGCAGCAACAGCAGCAGCAACTC  < DGRP 1.0/2.0
```

4. However, after aligning to the Sanger sequence for RAL-646, which we re-sequenced and found to contain a large gap, we find that mismatches are reduced to 56 differences from only 6 reads (previously was 67 differences from 10/13 reads). This represents a substantial improvement in alignment quality for a greater number of the original Illumina reads underlying DGRP assembly. With our new alignment, a total of seven reads have a perfect consensus.

```
REF.          1 CCCCAGCAACAGCAGCAGCAGCAGCAACAGCAACAGCAGCAACATCAGCAGCAGCAACAGCAGCAGCAACAGCAGCAGCAGCAACAGCAGCAGCAACTC
Sanger-646    * CCCCAGCAACAGCAGCAGCAGCAGCAGCAG------------------------CAACAGCAGCAGCAACAGCAGCAGCAGCAACAGCAGCAGCAACTC
2766069       0 CCCCAGCAACAGCAGCAGCAGCAGCAGCAG------------------------CA
2766082       0 CCCCAGCAACAGCAGCAGCAGCAGCAGCAG------------------------CAACAGCAGCAGC
2766083       1 CCCCAGCAACAGCAGCAGCAGCAGCAGCAG------------------------CAACAGCAGCAGCAAAAGCAGCAGCAGCAACA
2766075      24  CCACAAAAAGCACAAGAACAAGACACCGA------------------------CAACACCACCAGCAACAGCAGCAGCAGCAACAGCAGCAGCAACTC
2766076      10               CAACACCCCCCCGCG------------------------AAACAGCAGCAGCAACAGCAACAGCAGCAACAGCAGCAGCAACTC
2766089       0                 GCAGCAGCAGCAG------------------------CAACAGCAGCAGCAACAGCAGCAGCAGCAACAGCAGCAGCAACTC
2766072       8                 GCAGCAGCAACGC------------------------CACCGGCACCACCAACACCAGCAGCAGCAACAGCAGCAGCAACTC
2766087       0                    GCAGCAGCAG------------------------CAACAGCAGCAGCAACAGCAGCAGCAGCAACAGCAGCAGCAACTC
2766074      11                     CCCCCACCA------------------------CAACAGCACCACAACCAGCAGCAGCAGCAACAGCAGCAGCAACTC
2766092       0                                                              GCAGCAACAGCAGCAGCAGCAACAGCAGCAGCAACTC
2766078       1                                                                     CCGCAGCAGCAGCAACAGCAGCAGCAACTC
2766088       0                                                                       GCAGCAGCAGCAACAGCAGCAGCAACTC
2766090       0                                                                           CAGCAGCAACAGCAGCAGCAACTC
```

5. Thus, using the seven reads without mismatches to each other, which represent a majority of reads (7/13), we can reproduce the exact Sanger RAL-646 genotype. We thus conclude that opa23 reads were sequenced in the original RAL646 (DGRP 1.0) but were misassembled because large gaps are too prohibitive to search.

```
Sanger-646    * CCCCAGCAACAGCAGCAGCAGCAGCAGCAGCAACAGCAGCAGCAACAGCAGCAGCAGCAACAGCAGCAGCAACTC
2766069       0 CCCCAGCAACAGCAGCAGCAGCAGCAGCAGCA
2766082       0 CCCCAGCAACAGCAGCAGCAGCAGCAGCAGCAACAGCAGCAGC
2766089       0                  GCAGCAGCAGCAGCAACAGCAGCAGCAACAGCAGCAGCAGCAACAGCAGCAGCAACTC
2766087       0                     GCAGCAGCAGCAACAGCAGCAGCAACAGCAGCAGCAGCAACAGCAGCAGCAACTC
2766092       0                                       GCAGCAACAGCAGCAGCAGCAACAGCAGCAGCAACTC
2766088       0                                                GCAGCAGCAGCAACAGCAGCAGCAACTC
2766090       0                                                    CAGCAGCAACAGCAGCAGCAACTC
```

6. EXTRA: Illumina sequencing reads are 75 nucleotides long and a single read could have encompassed the 75 base pairs exactly underlying the "$PQ_{23}L$" coding sequence. We searched for Q_{23} reads or the central Q_{21}, Q_{19}, Q_{17}, or Q_{15} coding sequences to see if we could find reads that span the center poly-Q coding sequence without otherwise being anchored on the sides by non-polyQ encoding sequence. We did not find any such reads mapping to the *Notch* locus.
