# Supplementary material for: The Nature, Extent, and Consequences of Genetic Variation in the opa Repeats of Notch in Drosophila
Source: G3 (Bethesda). 2015 Sep 10;5(11):2405–19. doi: 10.1534/g3.115.021659 (PMC4632060; doi:10.1534/g3.115.021659)
Supplement: Supporting Information [file supp_g3.115.021659_FileS3.pdf]

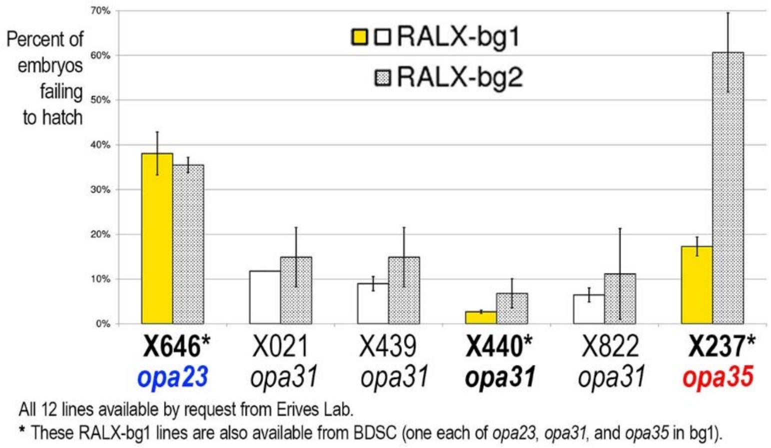

File S3 Figure showing the separate embryonic assay results for the RALX-bg1 and RALX-bg2 series.
